# Up-regulation of FOXD1 by YAP alleviates senescence and osteoarthritis

**DOI:** 10.1371/journal.pbio.3000201

**Published:** 2019-04-01

**Authors:** Lina Fu, Yuqiong Hu, Moshi Song, Zunpeng Liu, Weiqi Zhang, Fa-Xing Yu, Jun Wu, Si Wang, Juan Carlos Izpisua Belmonte, Piu Chan, Jing Qu, Fuchou Tang, Guang-Hui Liu

**Affiliations:** 1 National Laboratory of Biomacromolecules, CAS Center for Excellence in Biomacromolecules, Institute of Biophysics, Chinese Academy of Sciences, Beijing, China; 2 State Key Laboratory of Stem Cell and Reproductive Biology, Institute of Zoology, Chinese Academy of Sciences, Beijing, China; 3 University of Chinese Academy of Sciences, Beijing, China; 4 Beijing Advanced Innovation Center for Genomics, College of Life Sciences, Peking University, Beijing, China; 5 Biomedical Pioneering Innovation Center, Peking University, Beijing, China; 6 State Key Laboratory of Membrane Biology, Institute of Zoology, Chinese Academy of Sciences, Beijing, China; 7 Institute for Stem Cell and Regeneration, Chinese Academy of Sciences, Beijing, China; 8 Advanced Innovation Center for Human Brain Protection, National Clinical Research Center for Geriatric Disorders, Xuanwu Hospital Capital Medical University, Beijing, China; 9 Children's Hospital and Institutes of Biomedical Sciences, Fudan University, Shanghai, China; 10 Department of Molecular Biology, University of Texas Southwestern Medical Center, Dallas, Texas, United States of America; 11 Gene Expression Laboratory, Salk Institute for Biological Studies, La Jolla, California, United States of America; 12 Peking-Tsinghua Center for Life Sciences, Peking University, Beijing, China; 13 Ministry of Education Key Laboratory of Cell Proliferation and Differentiation, Beijing, China; 14 Beijing Institute for Brain Disorders, Beijing, China; Institute of Biochemistry and Cell Biology, Shanghai institutes for Biologic Sciences, Chinese Academy of Sciences, CHINA

## Abstract

Cellular senescence is a driver of various aging-associated disorders, including osteoarthritis. Here, we identified a critical role for Yes-associated protein (YAP), a major effector of Hippo signaling, in maintaining a younger state of human mesenchymal stem cells (hMSCs) and ameliorating osteoarthritis in mice. Clustered regularly interspaced short palindromic repeat (CRISPR)/CRISPR associated protein 9 nuclease (Cas9)-mediated knockout (KO) of YAP in hMSCs resulted in premature cellular senescence. Mechanistically, YAP cooperated with TEA domain transcriptional factor (TEAD) to activate the expression of forkhead box D1 (FOXD1), a geroprotective protein. YAP deficiency led to the down-regulation of FOXD1. In turn, overexpression of YAP or FOXD1 rejuvenated aged hMSCs. Moreover, intra-articular administration of lentiviral vector encoding YAP or FOXD1 attenuated the development of osteoarthritis in mice. Collectively, our findings reveal YAP–FOXD1, a novel aging-associated regulatory axis, as a potential target for gene therapy to alleviate osteoarthritis.

## Abstract 2

The Hippo signalling effector YAP and the transcription factor FOXD1 play a role in alleviating cellular senescence and osteoarthritis, identifying the YAP-FOXD1 axis as a potential therapeutic target for aging-associated disorders.

## Author summary

Stem cell aging contributes to aging-associated degenerative diseases. Studies aiming to characterize the mechanisms of stem cell aging are critical for obtaining a comprehensive understanding of the aging process and developing novel strategies to treat aging-related diseases. As a prevalent aging-associated chronic joint disorder, osteoarthritis is a leading cause of disability. Senescent mesenchymal stem cells (MSCs) residing in the joint may be a critical target for the prevention of osteoarthritis; however, the key regulators of MSC senescence are little known, and targeting aging regulatory genes for the treatment of osteoarthritis has not yet been reported. Here, we show that Yes-associated protein (YAP), a major effector of Hippo signaling, represses human mesenchymal stem cell (hMSC) senescence through transcriptional up-regulation of forkhead box D1 (FOXD1). Lentiviral gene transfer of YAP or FOXD1 can rejuvenate aged hMSCs and ameliorate osteoarthritis symptoms in mouse models. We propose that the YAP–FOXD1 axis is a novel target for combating aging-associated diseases.

## Introduction

Mesenchymal stem cells (MSCs) are widely distributed in adult tissues and have the capacities of self-renewal and differentiation into multiple cell lineages, such as chondrocytes, osteoblasts, and adipocytes [1]. MSCs are involved in tissue repair and homeostatic maintenance [2,3]. Over time, MSCs exhibit an age-associated decline in their number and function [4–6], namely, MSC senescence, which may be implicated in the loss of tissue homeostatic maintenance and leads to organ failure and degenerative diseases [7–10]. Therefore, an understanding of the mechanisms underlying MSC senescence will likely reveal novel therapeutic targets for ameliorating degenerative diseases.

Osteoarthritis is a prevalent aging-associated disorder that is characterized by the progressive deterioration of articular cartilage [11,12]. In osteoarthritis joints, degenerative changes start with cellular disorganization, gradual stiffening, and irregular surface of superficial zone followed by loss of matrix, clefts, and osteophyte formation in the deep articular cartilage [13,14]. Accordingly, disruption of the superficial zone of cartilage is an onset of osteoarthritis. Previous reports have demonstrated that cells isolated from the superficial zone of mouse and human articular cartilage express MSC markers, including cluster of differentiation (CD) 105, CD166, CD29, and exhibit MSC characteristics [15–20]. Cell death induced by oxidative stress or wound occurs primarily at the surface zone of cartilage [21,22]. When such cell death is inhibited by chemicals, cartilage disorganization and matrix loss are greatly reduced [23]. Therefore, MSCs or chondrocyte progenitor cells residing in the superficial zone of cartilage may be a critical target for the prevention of osteoarthritis. Although the transplantation of ex vivo cultures of MSCs into the osteoarthritic joint has been shown to improve the symptoms [24–26], the rejuvenation of endogenous senescent MSCs may also be a therapeutic option for osteoarthritis. The localized nature of osteoarthritis, which has no major extra-articular or systemic manifestations, makes it an ideal candidate for local, intra-articular gene therapy [27,28]. However, gene therapy strategies aiming at alleviating senescence, particularly MSC senescence, for treating osteoarthritis have not yet been reported.

Yes-associated protein (YAP) and transcriptional coactivator with PDZ-binding motif (TAZ) are primary targets of the Hippo signaling pathway, which plays important roles in the regulation of development, homeostasis, regeneration, and so forth [29–31]. The Hippo kinase cascade phosphorylates YAP and TAZ, resulting in their cytoplasmic retention and proteolytic degradation. When the Hippo pathway is inactive, YAP and TAZ translocate into the nucleus and interact with transcription factors to regulate the expression of target genes [32]. YAP and TAZ, as paralogs, have been demonstrated as key regulators in organ size control [33] and essential transducers of mechanical signals [34]. Here, we identified a critical role for YAP, but not TAZ, in regulating human MSC (hMSC) senescence. YAP exerted a geroprotective effect on hMSCs through the transcriptional activation of forkhead box D1 (*FOXD1)* in a TEA domain transcription factor (TEAD)-dependent manner. Gene therapy with lentiviral vectors encoding YAP or FOXD1 prevented cellular aging and attenuated osteoarthritis in mice. Our data suggest that YAP and its downstream target FOXD1 are novel suppressors of hMSC senescence and that the YAP–FOXD1 regulatory axis represents a potential therapeutic target for osteoarthritis.

## Results

### YAP, but not TAZ, safeguards hMSCs from senescence

**Fig 1:**
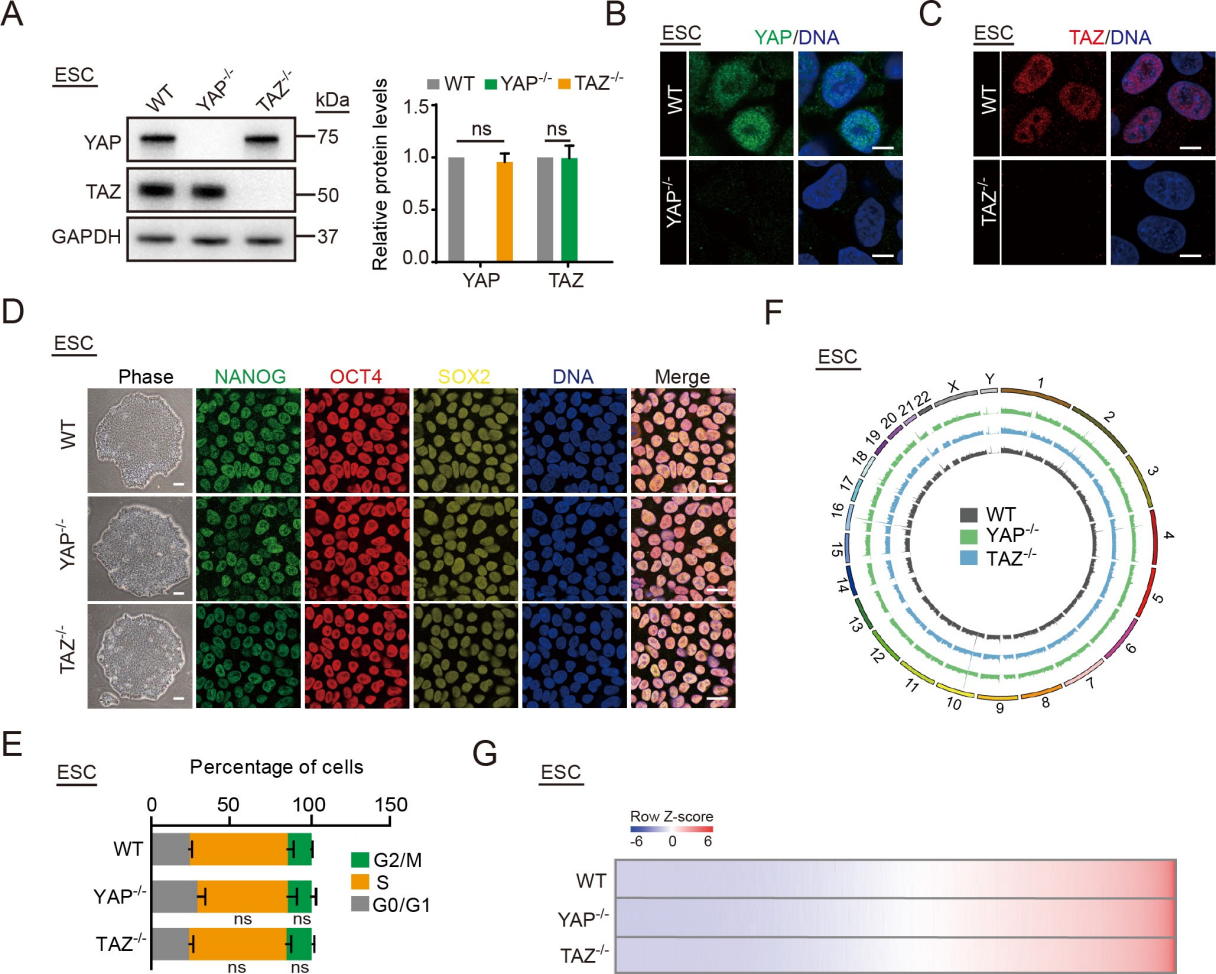
Generation of *YAP*^−/−^ and *TAZ*^−/−^ hESCs. (A) Western blot analysis of YAP and TAZ in *YAP*^−/−^ and *TAZ*^−/−^ hESCs. GAPDH was used as a loading control (left). The protein levels normalized with GAPDH were shown as fold change relative to WT hESCs (right). Data are presented as the mean ± SD, *n* = 3. (B) Immunostaining of YAP in WT and *YAP*^−/−^ hESCs. Scale bar, 10 μm. (C) Immunostaining of TAZ in WT and *TAZ*^−/−^ hESCs. Scale bar, 10 μm. (D) Immunofluorescence analysis of pluripotency markers NANOG, OCT4, and SOX2 in WT, *YAP*^−/−^, and *TAZ*^−/−^ hESCs. Scale bar of brightfield images is 200 μm; scale bar of immunofluorescence images is 25 μm. (E) Cell cycle analysis of WT, *YAP*^−/−^, and *TAZ*^−/−^ hESCs. Data are presented as mean ± SD, *n* = 3. (F) Whole-genome sequencing analysis of CNVs in WT, *YAP*^−/−^, and *TAZ*^−/−^ hESCs. (G) Heat map displaying the global transcriptomes of WT, *YAP*^−/−^, and *TAZ*^−/−^ hESCs. The numerical data underlying this figure are included in S8 Data. CNV, copy number variation; ESC, embryonic stem cell; G, gap; GAPDH, glyceraldehyde-3-phosphate dehydrogenase; hESC, human embryonic stem cell; M, mitosis; ns, not significant; OCT4, organic cation/carnitine transporter 4; S, synthesis; SOX2, SRY-box 2; TAZ, transcriptional coactivator with PDZ-binding motif; WT, wild type; YAP, Yes-associated protein.

We first used Clustered regularly interspaced short palindromic repeat (CRISPR)/CRISPR-associated protein 9 nuclease (Cas9)-mediated gene editing [35] to generate isogenic human embryonic stem cells (hESCs) lacking *YAP* or *TAZ* to study the functions of YAP and TAZ in regulating human stem cell homeostasis (S1A, S1B and S1G Fig). Successful gene targeting at the *YAP* or *TAZ* locus were verified by genomic polymerase chain reaction (PCR) and reverse transcription quantitative PCR (RT-qPCR) (S1C and S1D Fig). Western blot and immunofluorescence further confirmed the complete ablation of YAP and TAZ protein in *YAP*^−/−^ and *TAZ*^−/−^ hESCs, respectively, with no detectable compensations between YAP and TAZ (Fig 1A, 1B and 1C). Both *YAP*^−/−^ and *TAZ*^−/−^ hESCs maintained normal pluripotency (Fig 1D) and cell cycle kinetics (Fig 1E) and were able to differentiate into tissues composed of all 3 germ layers in vivo (S1E Fig). Karyotype and genome-wide copy number variation (CNV) analyses demonstrated that genomic integrity was maintained in *YAP*^−/−^ and *TAZ*^−/−^ hESCs after more than 30 passages (Figs 1F and S1F). Moreover, *YAP*^−/−^ and *TAZ*^−/−^ hESCs displayed transcriptional profiles that were highly similar to wild type (WT) hESCs (Fig 1G).

**Fig 2:**
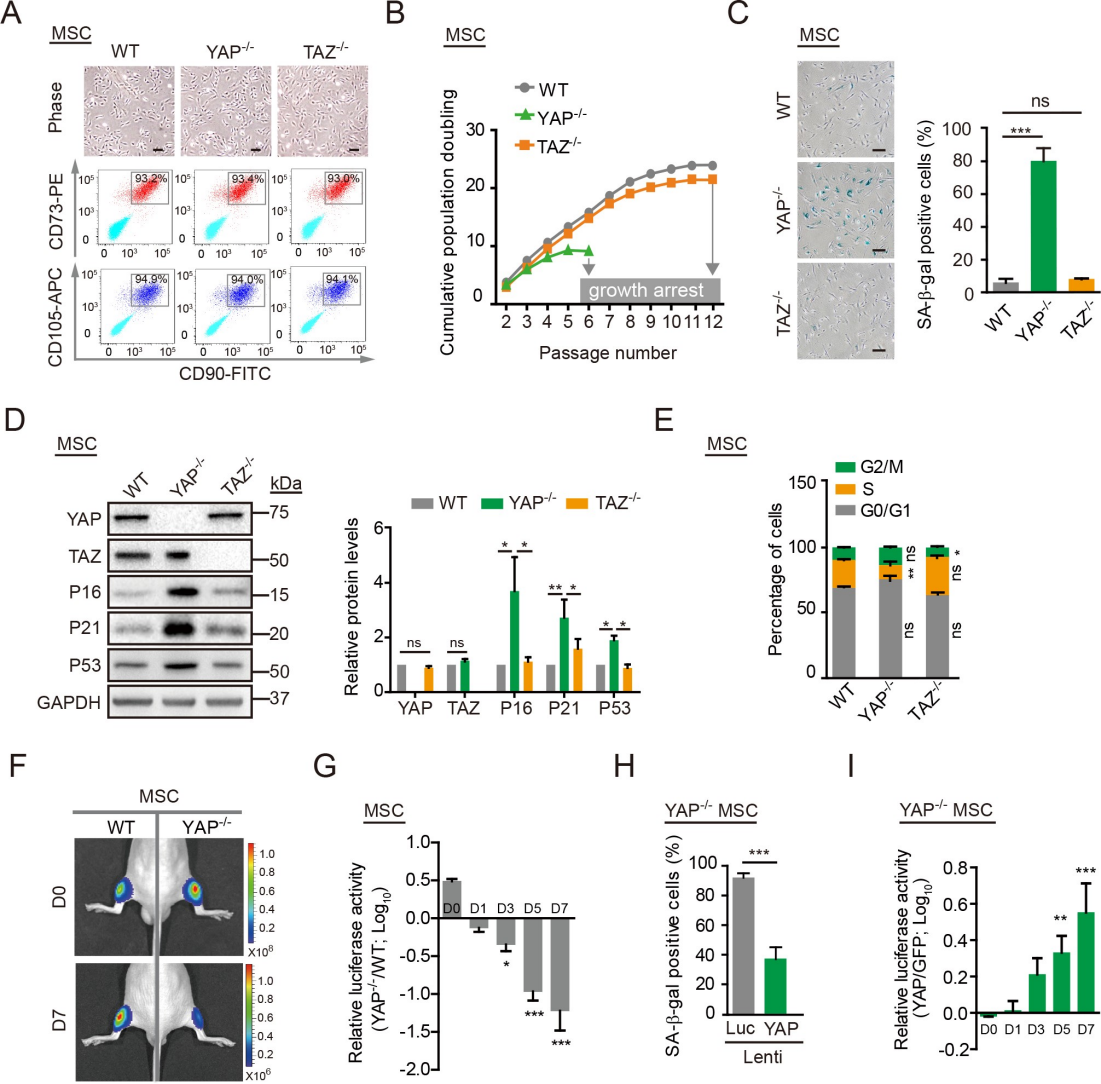
*YAP*^−/−^ hMSCs exhibit accelerated senescence. (A) Brightfield micrographs and FACS analysis of the surface markers CD105, CD73, and CD90 in WT, *YAP*^−/−^, and *TAZ*^−/−^ hMSCs. Scale bar, 50 μm. (B) Cell growth curves of WT, *YAP*^−/−^, and *TAZ*^−/−^ hMSCs. Data are presented as the mean ± SD, *n* = 3. (C) SA-β-gal staining of WT, *YAP*^−/−^, and *TAZ*^−/−^ hMSCs at passage 4. Scale bar, 100 μm. Data are presented as the mean ± SD, *n* = 3, ***P < 0.001. (D) Western blot analysis of YAP, TAZ, P16, P21, and P53 in WT, *YAP*^−/−^, and *TAZ*^−/−^ hMSCs. GAPDH was used as a loading control (left). The protein levels normalized with GAPDH were shown as fold change relative to WT hMSCs (right). Data are presented as the mean ± SD, *n* = 3, *P < 0.05, **P < 0.01. (E) Cell cycle analysis of WT, *YAP*^−/−^, and *TAZ*^−/−^ hMSCs. Data are presented as the mean ± SD, *n* = 3, *P < 0.05, **P < 0.01. (F) WT and *YAP*^−/−^ hMSCs transduced with a lentivirus expressing Luc were injected into the TA muscle of immunodeficient mice. Luc activities were imaged at day (D)0, D1, D3, D5, and D7 after cell implantation. Representative images at D0 and D7 are shown. (G) Data are presented as the ratios of *YAP*^−/−^ to WT cells (log_10_ (fold)), mean ± SD, *n* = 5, *P < 0.05, ***P < 0.001. (H) SA-β-gal staining of *YAP*^−/−^ hMSCs transduced with lentiviruses expressing Luc or YAP. Data are presented as the mean ± SD, *n* = 3, ***P < 0. 001. (I) *YAP*^−/−^ hMSCs overexpressing GFP plus Luc and *YAP*^−/−^ hMSCs overexpressing YAP plus Luc were implanted into the TA muscles of immunodeficient mice. The Luc activities were measured at different time points after cell implantation. Data are presented as the ratios of YAP to GFP (log_10_ (fold)), mean ± SD, *n* = 5, **P < 0.01, ***P < 0.001. The numerical data underlying this figure are included in S8 Data. APC, allophycocyanin; CD, cluster of differentiation; D, day; FACS, fluorescence activated cell sorter; FITC, fluorescein isothiocyanate; G, gap; GAPDH, glyceraldehyde-3-phosphate dehydrogenase; GFP, green fluorescent protein; hMSC, human mesenchymal stem cell; Lenti, lentivirus; Luc, luciferase; M, mitosis; MSC, mesenchymal stem cell; ns, not significant; PE, phycoerythrin; S, synthesis; SA-β-gal, senescence-associated-β-galactosidase; TA, tibialis anterior; TAZ, transcriptional coactivator with PDZ-binding motif; WT, wild type; YAP, Yes-associated protein.

We next differentiated WT, *YAP*^−/−^, and *TAZ*^−/−^ hESCs into hMSCs (Fig 2A) [35–38]. The derived hMSCs expressed a series of hMSC markers including CD105, CD166, CD29, CD90, CD73, CD44, CD13, and human leukocyte antigens, A, B and C (HLA-ABC) and were negative for hematopoietic or skeletal lineage markers CD34, CD43, CD45, CD14, CD19, podoplanin (PDPN), and CD164, resembling the resident CD105^+^, CD166^+^, and CD29^+^ MSCs in the superficial zone of articular cartilage (Figs 2A and S2A) [39–41]. Whereas WT hMSCs were able to differentiate into chondrocytes, osteoblasts, and adipocytes, *YAP*^−/−^ and *TAZ*^−/−^ hMSCs exhibited compromised differentiation abilities into osteoblasts and chondrocytes (S2B–S2E Fig). Additionally, the growth rates of WT, *YAP*^−/−^, and *TAZ*^−/−^ hMSCs were analyzed through in vitro serial passaging. Compared with WT and *TAZ*^−/−^ hMSCs, YAP-deficient hMSCs exhibited early-onset aging characteristics and arrested at passage 6 (Fig 2B). Increased levels of senescence-associated–β-galactosidase (SA-β-gal) activity (Fig 2C) and increased expression of P16, P53, and P21 (Fig 2D) were detected at as early as passage 4. Concomitantly, *YAP*^−/−^ hMSCs showed a series of premature phenotypes, including (1) a decreased percentage of cells in synthesis (S) phase and increased percentages of cells in gap phases (G0, G1, and G2) and mitosis (M) phase (Fig 2E), (2) a lower percentage of Ki67-positive cells (S2F and S2G Fig), (3) decreased levels of the nuclear lamina-associated protein 2 (LAP2; S2F and S2G Fig), (4) reduced levels of heterochromatin protein 1 alpha (HP1α) and heterochromatin protein 1 gamma (HP1γ; S2F and S2G Fig), and (5) higher levels of reactive oxygen species (ROS; S3A Fig), compared with WT and *TAZ*^−/−^ hMSCs. We subsequently examined whether the YAP deficiency resulted in stem cell attrition in vivo. WT, *YAP*^−/−^, and *TAZ*^−/−^ hMSCs were transduced with a lentiviral vector expressing luciferase (Luc) and injected into the tibialis anterior (TA) muscles of immunodeficient mice. Consistent with the in vitro observations, *YAP*^−/−^ hMSCs, but not *TAZ*^−/−^ or WT hMSCs, exhibited an accelerated functional decay after transplantation in vivo (Figs 2F and 2G and S3B and S3C).

To further evaluate the effect of YAP in hMSCs, WT hMSCs were transduced with lentiviruses encoding a single guide RNA (sgRNA) targeting *YAP* or a non-targeting control (NTC) sgRNA, as well as CRISPR/Cas9 [42,43]. Phenotypic characterizations revealed that the down-regulation of YAP in hMSCs also resulted in a similar premature aging phenotype (S3D and S3E Fig). By contrast, ectopic expression of YAP rescued the premature senescence observed in *YAP*^−/−^ hMSCs, as evidenced by the reduced percentage of SA-β-gal–positive cells (Fig 2H), enhanced growth rate and clonal expansion ability (S3F and S3G Fig), decreased expression of P16 and P21 (S3H Fig), lower levels of ROS (S3I Fig), and slower in vivo decay after engraftment (Figs 2I and S3J). Taken together, these data suggest that YAP, but not TAZ, plays an essential role in protecting hMSCs from premature senescence.

### YAP suppresses hMSC senescence in a TEAD-dependent manner

**Fig 3:**
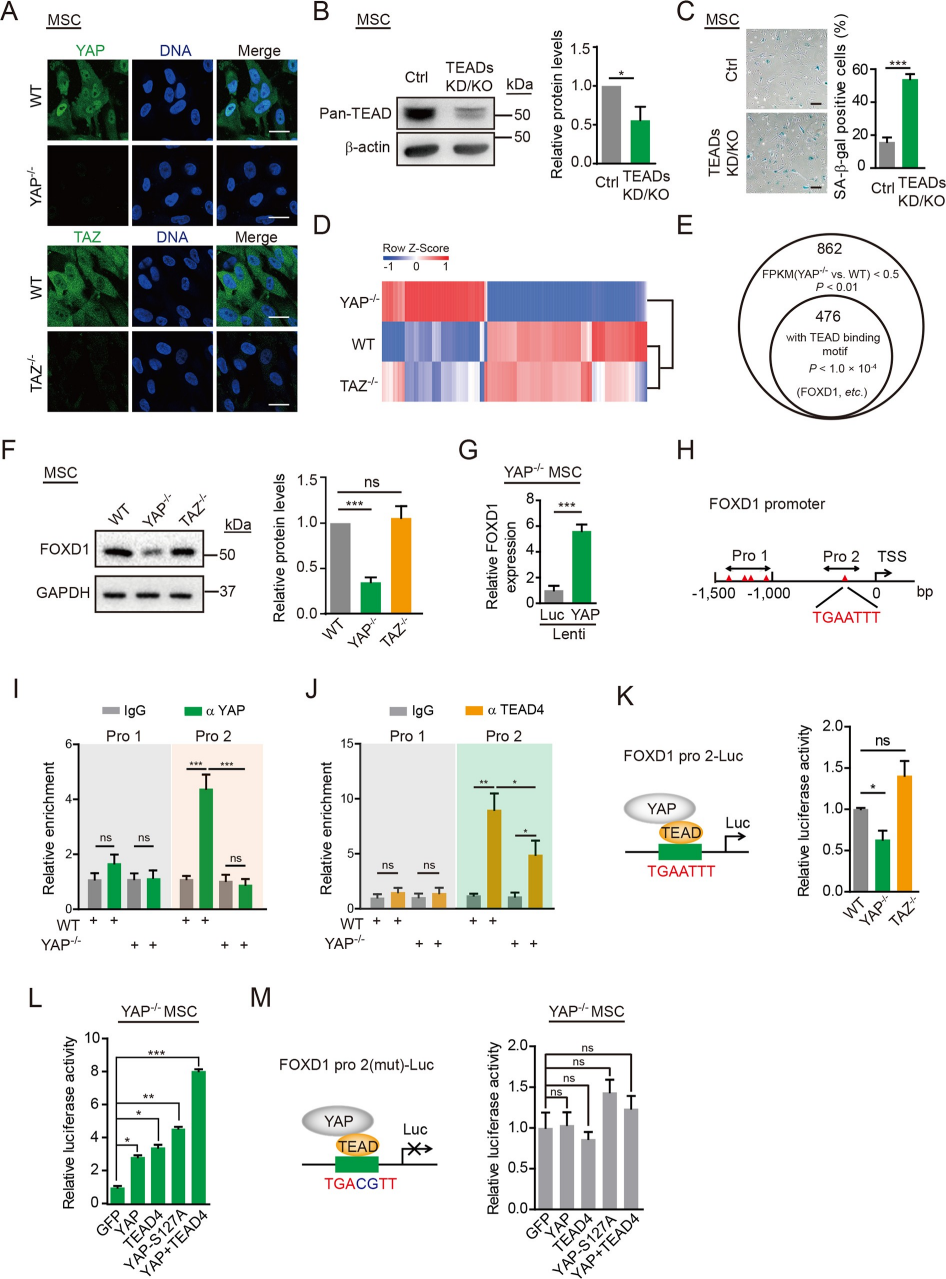
YAP transcriptionally induces *FOXD1* expression. (A) Immunofluorescence analysis of YAP in WT and *YAP*^−/−^ hMSCs, and TAZ in WT and *TAZ*^−/−^ hMSCs. Scale bar, 25 μm. (B) Western blot analysis of pan-TEAD proteins in Ctrl and TEADs KD/KO hMSCs. β-actin was used as a loading Ctrl (left). The protein levels normalized with β-actin were shown as fold change relative to Ctrl hMSCs (right). Data are presented as the mean ± SD, *n* = 3, *P < 0.05. (C) SA-β-gal staining of Ctrl and TEADs KD/KO hMSCs. Scale bar, 100 μm. Data are presented as the mean ± SD, *n* = 3, ***P < 0.001. (D) A heat map showing relative mRNA expression levels of the differentially expressed genes in *YAP*^−/−^ hMSCs. Genes were sorted by the fold change and *P* value (fold change > 2 or < 0.5, *P* < 0.01). Corresponding gene expression profiles obtained from *TAZ*^−/−^ hMSCs were also shown. (E) The bioinformatics analysis predicted that 476 (55%) of 862 genes down-regulated in *YAP*^−/−^ hMSCs were potential YAP–TEAD targets. Among these genes, *FOXD1* was the most down-regulated gene. (F) Western blot analysis of FOXD1 in WT, *YAP*^−/−^, and *TAZ*^−/−^ hMSCs. GAPDH was used as a loading Ctrl (left). The protein levels normalized with GAPDH were shown as fold change relative to WT hMSCs (right). Data are presented as the mean ± SD. *n* = 3, ***P < 0.001. (G) RT-qPCR showing elevated expression of *FOXD1* in *YAP*^−/−^ hMSCs transduced with a lentivirus encoding YAP. Data are presented as the mean ± SD, *n* = 3, ***P < 0.001. (H) TEAD binding sites were examined in the *FOXD1* Pro, and the putative binding sites are depicted as triangles. (I) ChIP-qPCR for YAP enrichment within different *FOXD1* Pro regions (Pro1 and Pro 2) containing putative TEAD binding motifs. Data are presented as the mean ± SD, *n* = 3, ***P < 0.001. (J) ChIP-qPCR for TEAD4 enrichment within different *FOXD1* Pro regions (Pro1 and Pro 2) containing putative TEAD binding motifs. Data are presented as the mean ± SD, *n* = 3, *P < 0.05, **P < 0.01. (K) The *FOXD1* Pro region (Pro 2) was cloned upstream of a Luc reporter, and Luc activity was detected in WT, *YAP*^−/−^, and *TAZ*^−/−^ hMSCs. Data are presented as the mean ± SD, *n* = 3, *P < 0.05. (L) The *FOXD1* Pro-mediated Luc activity was detected in *YAP*^−/−^ hMSCs in the indicated experiments. Data are presented as the mean ± SD, *n* = 3, *P < 0.05, **P < 0.01, ***P < 0.001. (M) The mutant *FOXD1* Pro2 driven Luc activity was detected in the indicated experiments. Data are presented as the mean ± SD, *n* = 3. The numerical data underlying this figure are included in S8 Data. ChIP, chromatin immunoprecipitation; Ctrl, control; FOXD1, forkhead box D1; FPKM, fragments per kilobase per million mapped fragments; GAPDH, glyceraldehyde-3-phosphate dehydrogenase; GFP, green fluorescent protein; hMSC, human mesenchymal stem cell; IgG, Immunoglobulin G; KD, knockdown; KO, knockout; Luc, luciferase; MSC, mesenchymal stem cell; mut, mutant; ns, not significant; Pro, promoter; RT-qPCR, reverse transcription quantitative polymerase chain reaction; S127A, serine127alanine; SA-β-gal, senescence-associated-β-galactosidase; TAZ, transcriptional coactivator with PDZ-binding motif; TEAD, TEA domain transcriptional factor; TSS, transcriptional start site; WT, wild type; YAP, Yes-associated protein.

Given that YAP and TAZ displayed distinct functions in regulating hMSC senescence, we next examined whether there were differences in the subcellular localizations of YAP and TAZ. In hMSCs, YAP was predominantly located in the nucleus, whereas TAZ was in the cytoplasm (Fig 3A). It has been shown that nuclear YAP binds transcription factors, including TEAD family of transcription factors (TEAD1, 2, 3, and 4), as a transcriptional coactivator to induce target gene expression and thus regulate a series of cellular processes [44]. To test whether the nuclear YAP acted in conjunction with TEAD to regulate hMSC senescence, we blocked the activities of all the members of TEAD family in hMSCs as confirmed by immunoblotting analysis (referred to as TEADs knockdown [KD] and KO hMSCs, TEADs KD/KO hMSCs; Fig 3B). Similar to YAP-deficient hMSCs, TEADs KD/KO hMSCs also showed major phenotypes of premature senescence, such as an increased number of SA-β-gal–positive cells (Fig 3C), compromised clonal expansion abilities (S4A Fig), and up-regulation of P16 and P21 (S4B Fig). These observations suggest that YAP safeguards hMSCs from premature senescence in a TEAD-dependent manner.

### FOXD1 mediates YAP deficiency-induced senescence

To elucidate the molecular mechanism underlying YAP–TEAD regulation of hMSC senescence, RNA sequencing (RNA-seq) analyses of WT, *YAP*^−/−^, and *TAZ*^−/−^ hMSCs were performed (S4C Fig). *TAZ*^−/−^ hMSCs displayed comparable transcriptional features compared to those of WT hMSCs, whereas *YAP*^−/−^ hMSCs exhibited a substantial number of differentially expressed genes (Figs 3D and S4E and S2 and S3 Datas). We observed few overlaps between differentially expressed genes in YAP KO and TAZ KO hMSCs compared to WT cells (S4D Fig), consistent with differential subcellular localization patterns of YAP and TAZ in hMSCs. Searching for TEAD binding motifs in the genome [45,46] identified 476 (55%) of the 862 down-regulated genes in *YAP*^−/−^ hMSCs as potential TEAD targets (*P* < 1.0 × 10^−4^; Fig 3E). Among them, *FOXD1* was the most significantly down-regulated gene in *YAP*^−/−^ hMSCs (S4 Data).

Western blotting verified the down-regulation of FOXD1 expression in *YAP*^−/−^ hMSCs (Fig 3F) as well as its up-regulation upon the reintroduction of YAP (Fig 3G), suggesting that *FOXD1* was transcriptionally controlled by YAP. We examined the *FOXD1* promoter region, including 1,500 bp upstream of the transcriptional start site (TSS) and identified 4 putative TEAD binding sites between −1,500 and −1,000 bp and 1 between −1,000 bp and the TSS (Fig 3H). Accordingly, we detected these 2 regions followed by chromatin immunoprecipitation (ChIP) using YAP and TEAD4 antibodies, revealing that YAP and TEAD4 bound predominantly within 1,000 bp upstream of the *FOXD1* TSS, where there was a putative TEAD binding site (Fig 3I and 3J). Next, we cloned this promoter region (−1,000 bp to the TSS) as a transcriptional element upstream of a basic Luc reporter. Reporter activity was lower in *YAP*^−/−^ hMSCs than in WT cells (Fig 3K) and was increased upon YAP or TEAD4 overexpression. Luc activity was even higher upon the expression of a constitutively activated YAP mutant (YAP-S127A) and was further enhanced by coexpression of YAP and TEAD4 (Fig 3L). The high levels of Luc activity were significantly abolished when we mutated the predicted TEAD binding site (Fig 3M). By contrast, ChIP assay demonstrated that TAZ did not bind to the *FOXD1* promoter (S4F Fig), and the Luc activity was insensitive to cellular TAZ levels (Figs 3K and S4G). Therefore, the YAP–TEAD pathway, but not TAZ, transcriptionally activates *FOXD1* expression.

**Fig 4:**
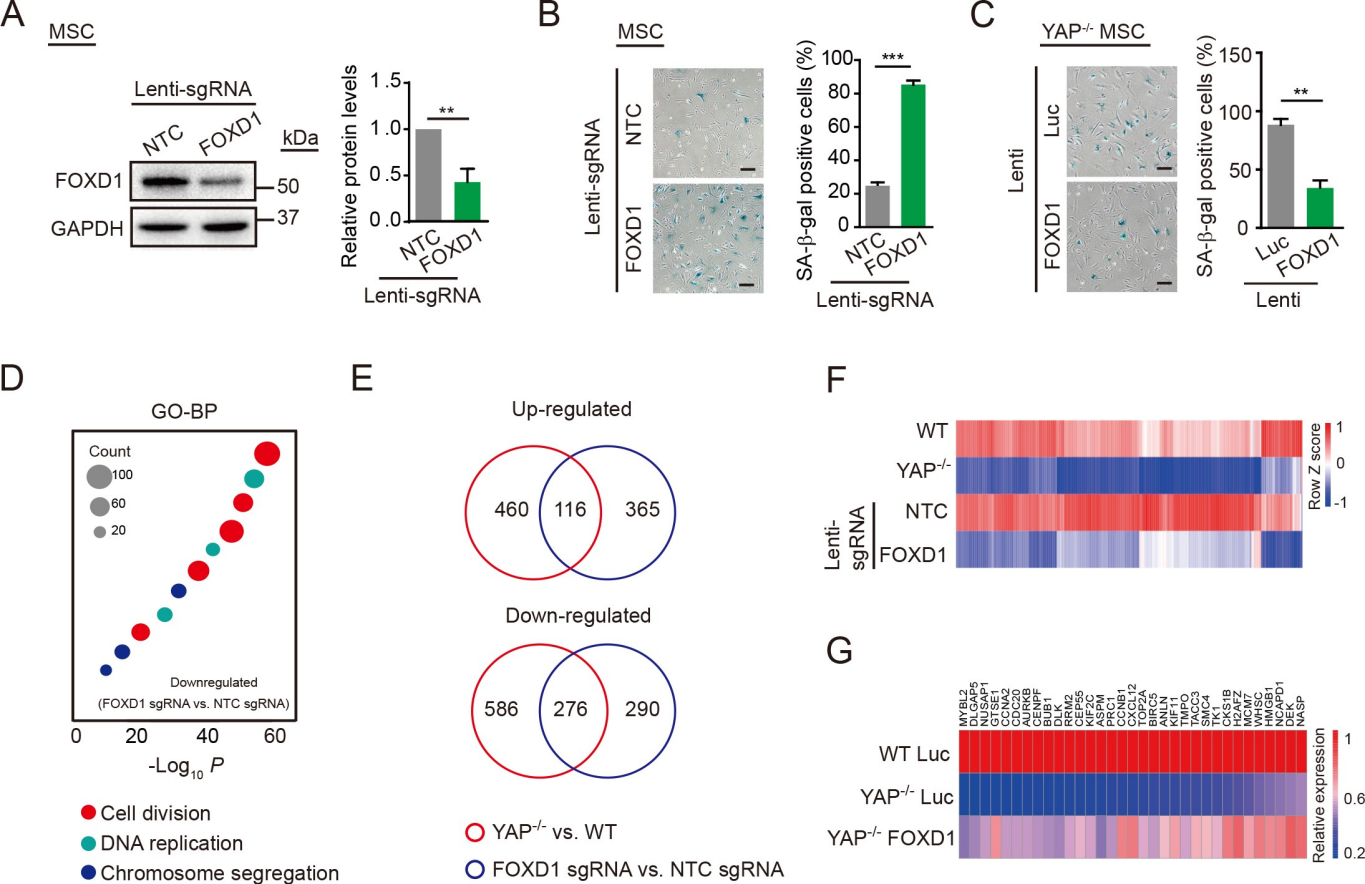
FOXD1 mediates YAP deficiency-induced senescence. (A) Western blot analysis of FOXD1 in hMSCs transduced with lentiviruses expressing NTC or *FOXD1* sgRNA, as well as CRISPR/Cas9. GAPDH was used as a loading control (left). The protein levels normalized with GAPDH were shown as fold change relative to lenti-NTC sgRNA transduced hMSCs (right). Data are presented as the mean ± SD, *n* = 3, **P < 0.01. (B) SA-β-gal staining of hMSCs transduced with lentiviruses expressing NTC or *FOXD1* sgRNA, as well as CRSPR/Cas9. Scale bar, 100 μm. Data are presented as the mean ± SD, *n* = 3, ***P < 0.001. (C) SA-β-gal staining of *YAP*^−/−^ hMSCs transduced with lentiviruses expressing Luc or FOXD1. Scale bar, 100 μm. Data are presented as the mean ± SD, *n* = 3, **P < 0.01. (D) GO-BP enrichment analysis of down-regulated genes in FOXD1 KO hMSCs. (E) Venn diagram showing differentially expressed genes in both *YAP*^−/−^ hMSCs (relative to WT) and FOXD1 KO hMSCs (relative to NTC). (F) RNA-seq heat map of the genes that were down-regulated both in *YAP*^−/−^ and FOXD1 KO hMSCs. (G) RT-qPCR heat map showing the rescue of aging-associated genes by FOXD1 overexpression in *YAP*^−/−^ hMSCs. The numerical data underlying this figure are included in S8 Data. ANLN, anillin actin binding protein; ASPM, abnormal spindle microtubule assembly; AURKB, aurora kinase B; BIRC5, baculoviral IAP repeat containing 5; BP, biological process; BUB1, BUB1 mitotic checkpoint serine/threonine kinase; Cas9, CRISPR associated protein 9 nuclease; CCNA2, cyclin A2; CCNB1, cyclin B1; CDC20, cell division cycle 20; CENPF, centromere protein F; CEP55, centrosomal protein 55; CKS1B, CDC28 protein kinase regulatory subunit 1B; CRISPR, Clustered Regularly Interspaced Short Palindromic Repeats; CXCL12, C-X-C motif chemokine ligand 12; DEK, DEK proto-oncogene; DLGAP5, DLG associated protein 5; DLK, delta like non-canonical Notch ligand 1; FOXD1, forkhead box D1; GAPDH, glyceraldehyde-3-phosphate dehydrogenase; GTSE1, G2 and S-phase expressed 1; GO, gene ontology; H2AFZ, H2A histone family member Z; HMGB1, high mobility group box 1; hMSC, human mesenchymal stem cell; KIF2C, kinesin family member 2C; KIF11, kinesin family member 11; KO, knockout; lenti, lentivirus; Luc, luciferase; MCM7, minichromosome maintenance complex component 7; MYBL2, MYB proto-oncogene like 2; MSC, mesenchymal stem cell; ns, not significant; NASP, nuclear autoantigenic sperm protein; NUSAP1, nucleolar and spindle associated protein 1; NTC, non-targeting control; PRC1, protein regulator of cytokinesis 1; RNA-seq, RNA sequencing; RRM2, ribonucleotide reductase regulatory subunit M2; RT-qPCR, reverse transcription quantitative polymerase chain reaction; SA-β-gal, senescence-associated-β-galactosidase; sgRNA, single guide RNA; SMC4, structural maintenance of chromosomes 4; TACC3, transforming acidic coiled-coil containing protein 3; TK1, thymidine kinase 1; TMPO, thymopoietin; TOP2A, DNA topoisomerase II alpha; WT, wild type; YAP, Yes-associated protein.

FOXD1 was initially implicated in renal development [47], but there was a lack of evidence for a link between FOXD1 and cellular senescence. To investigate whether FOXD1 participated in YAP deficiency-induced accelerated senescence of hMSCs, we knocked out FOXD1 in hMSCs using a lentiviral vector-dependent CRISPR/Cas9 system [42,43] (Figs 4A and S5A). FOXD1 depletion in hMSCs increased the percentage of SA-β-gal–positive cells (Fig 4B), inhibited clonal expansion (S5B Fig), and up-regulated P16 and P21 expression (S5C Fig), recapitulating the major phenotypes implicated in premature senescence caused by the YAP deficiency. The overexpression of FOXD1 in *YAP*^−/−^ hMSCs effectively alleviated the accelerated senescence (Fig 4C). In addition, we also examined the gene expression profile of FOXD1 KO hMSCs using RNA-seq (S5 Data). FOXD1 KO decreased the expression of genes that were mainly associated with cell division and DNA replication, which ultimately contributed to the senescence phenotypes (Fig 4D and S6 Data). Combined analyses with WT and *YAP*^−/−^ hMSCs showed that FOXD1-deficient and *YAP*^−/−^ hMSCs were similar to each other at the transcriptomic level (S5D and S5E Fig). Many differentially expressed genes were overlapped between *YAP*^−/−^ (compared to WT) and FOXD1 KO (compared to NTC-transduced) hMSCs, including 116 up-regulated genes accounting for 20% of the total up-regulated genes in *YAP*^−/−^ hMSCs and 276 down-regulated genes accounting for 32% of the total down-regulated genes in *YAP*^−/−^ hMSCs (Fig 4E and 4F), implying an important role for FOXD1 in mediating YAP deficiency-induced premature cellular aging. Of note, many of those commonly down-regulated genes were elevated upon ectopic expression of FOXD1 in *YAP*^−/−^ hMSCs (Fig 4G). Conversely, ectopic expression of YAP in FOXD1 KO or TEADs KD/KO hMSCs did not exert obvious rescue effect on the senescence phenotypes (S5F and S5G Fig). Taken together, these data indicate that down-regulation of FOXD1, an effector of YAP–TEAD signaling, contributes to the premature senescence induced by YAP deficiency.

### The YAP–FOXD1 axis exerts a geroprotective effect on replicative and pathological cellular aging

**Fig 5:**
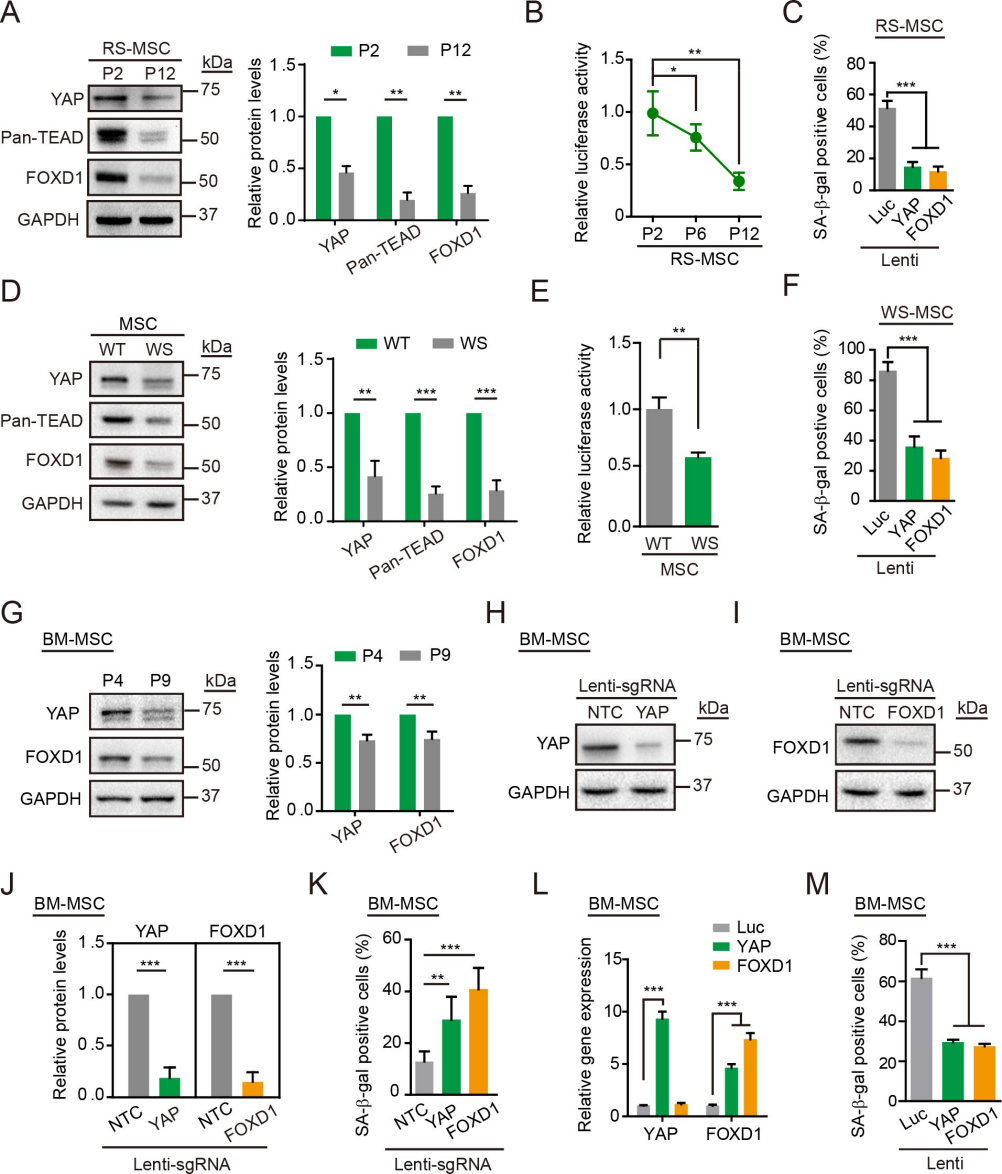
The YAP–FOXD1 axis counteracts replicative and pathological senescence. (A) Western blot analysis of YAP, Pan-TEAD, and FOXD1 in RS hMSCs. GAPDH was used as a loading control. The protein levels normalized with GAPDH were shown as fold change relative to P2 hMSCs (right). Data are presented as the mean ± SD, *n* = 3, *P < 0.05, **P < 0.01. (B) The 8 × GTIIC-Luc activity detected in RS hMSCs. Data are presented as the mean ± SD, *n* = 3, *P < 0.05, **P < 0.01. (C) SA-β-gal staining of RS hMSCs transduced with lentiviruses expressing Luc, YAP, or FOXD1. Data are presented as the mean ± SD, *n* = 3, ***P < 0.001. (D) Western blot analysis of YAP, Pan-TEAD, and FOXD1 in WT and WS hMSCs. GAPDH was used as a loading control (left). The protein levels normalized with GAPDH were shown as fold change relative to WT hMSCs (right). Data are presented as the mean ± SD, *n* = 3, **P < 0.01, ***P < 0.001. (E) The 8 × GTIIC-Luc activity determined in WT and WS hMSCs. Data are presented as the mean ± SD, *n* = 3, **P < 0.01. (F) SA-β-gal staining of WS hMSCs transduced with lentiviruses expressing Luc, YAP, or FOXD1. Data are presented as the mean ± SD, *n* = 3, ***P < 0.001. (G) Western blot analysis of YAP and FOXD1 in RS BM-hMSCs. GAPDH was used as a loading control (left). The protein levels normalized with GAPDH were shown as fold change relative to P4 BM-hMSCs (right). Data are presented as the mean ± SD, *n* = 3, **P < 0.01. (H) Western blot analysis of YAP in BM-hMSCs transduced with lentiviruses expressing NTC or *YAP* sgRNA as well as CRISPR/Cas9. GAPDH was used as a loading control. (I) Western blot analysis of FOXD1 in BM-hMSCs transduced with lentiviruses expressing NTC or *FOXD1* sgRNA. GAPDH was used as a loading control. (J) The protein levels normalized with GAPDH were shown as fold change relative to lenti-NTC sgRNA transduced BM-hMSCs. Data are presented as the mean ± SD, *n* = 3, ***P < 0.001. (K) SA-β-gal staining of BM-hMSCs transduced with lentiviruses expressing NTC, *YAP*, or *FOXD1* sgRNA as well as CRISPR/Cas9. Data are presented as the mean ± SD, *n* = 3, **P < 0.01, ***P < 0.001. (L) RT-qPCR detection of *YAP* and *FOXD1* expression levels in BM-hMSCs transduced with lentiviruses expressing Luc, YAP, or FOXD1. Data are presented as the mean ± SD, *n* = 3, ***P < 0.001. (M) SA-β-gal staining of BM-hMSCs transduced with lentiviruses expressing Luc, YAP, or FOXD1. Data are presented as the mean ± SD, *n* = 3, ***P < 0.001. The numerical data underlying this figure are included in S8 Data. BM-hMSC, human mesenchymal stem cells isolated from human bone marrow; Cas9, CRISPR associated protein 9 nuclease; CRISPR, Clustered Regularly Interspaced Short Palindromic Repeats; FOXD1, forkhead box D1; GAPDH, glyceraldehyde-3-phosphate dehydrogenase; hMSC, human mesenchymal stem cell; lenti, lentivirus; Luc, luciferase; ns, not significant; NTC, non-targeting control; RS, replicative-senescent; RT-qPCR, reverse transcription quantitative polymerase chain reaction; SA-β-gal, senescence-associated-β-galactosidase; sgRNA, single guide RNA; TEAD, TEA domain transcriptional factor; WS, Werner syndrome; WT, wild type; YAP, Yes-associated protein.

To further elucidate the relationship between the YAP–FOXD1 axis and human stem cell aging, we examined the expression levels of YAP, pan-TEAD, and FOXD1 in both replicative-senescent (RS) hMSCs and Werner syndrome (WS) hMSCs, a human stem cell model for premature aging disorder WS [37,48]. Western blotting revealed decreased levels of YAP, pan-TEAD, and FOXD1 in both types of senescent hMSCs (Fig 5A and 5D). Moreover, the activity of 8 × GTIIC-Luc, a YAP/TAZ-responsive reporter, decreased in both RS hMSCs and WS hMSCs (Fig 5B and 5E). Lentiviral overexpression of YAP or FOXD1 effectively attenuated the senescent features of RS hMSCs (Figs 5C and S6A–S6C) and WS hMSCs (Figs 5F and S6D–S6G). We also observed diminished protein levels of YAP and FOXD1 in RS-primary hMSCs isolated from human bone marrow (BM-hMSCs) (Fig 5G). In BM-hMSCs, KO of YAP or FOXD1 with a CRISPR/Cas9 system promoted cellular senescence (Fig 5H–5K), whereas the overexpression of YAP or FOXD1 delayed BM-hMSC senescence (Fig 5L and 5M). Collectively, these observations establish a geroprotective role for the YAP–FOXD1 axis in alleviating hMSC aging.

### Overexpression of YAP or FOXD1 attenuates post-traumatic osteoarthritis in mice

Mesodermal cellular aging has emerged as a fundamental hallmark of aging-related disorders, including osteoarthritis, one of the most common degenerative diseases, the incidence of which increases significantly with age. Dysfunction of MSCs residing in the superficial zone of cartilage precedes osteoarthritis [15–19,21,22] that is characterized by articular cartilage degradation [49–51]. To validate a role of hMSC senescence in driving osteoarthritis, we injected young hMSCs, RS hMSCs, and RS hMSCs overexpressing YAP or FOXD1, respectively, into the joints of immunodeficient mice and performed histological assessment of the joints 1 month later (S7A Fig). In line with a previous report [52], Safranin O staining revealed the delamination of the articular surface and erosion of articular cartilage in the RS hMSC–administrated joints (S7B and S7C Fig). However, no osteoarthritis-related features manifested in the joints transplanted with young hMSCs or RS hMSCs overexpressing YAP or FOXD1. RT-qPCR further demonstrated that RS hMSCs, rather than young hMSCs, and RS hMSCs overexpressing YAP or FOXD1 induced aging markers in mouse joints (S7D Fig). These results suggest that accumulation of senescent MSCs in joints contributes to the development of osteoarthritis, which can be eliminated by YAP or FOXD1 overexpression.

**Fig 6:**
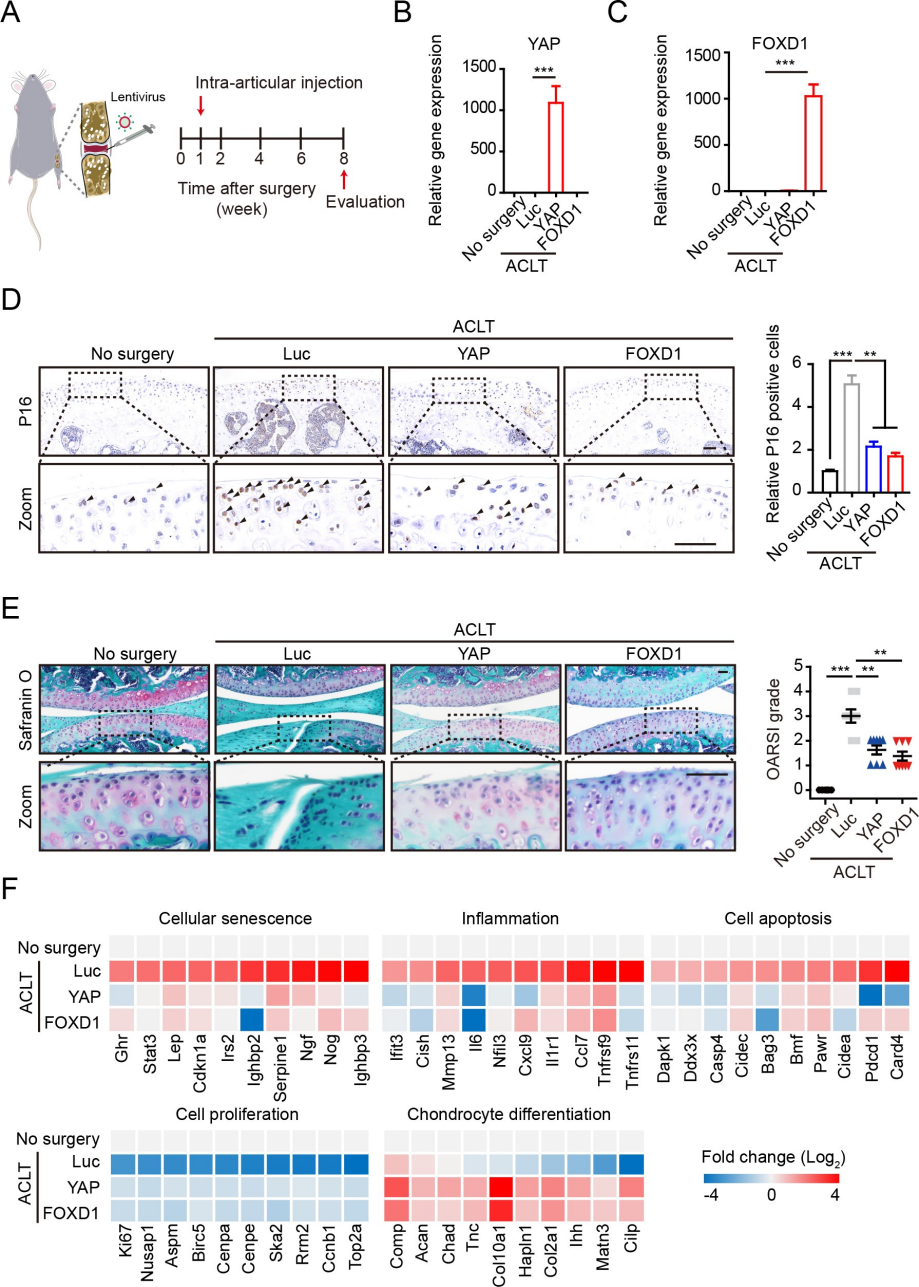
The YAP and FOXD1 gene therapy alleviates osteoarthritis in mice. (A) Schematic of the time course used for the in vivo osteoarthritis experiments. (B and C) RT-qPCR analysis of YAP and FOXD1 expression in the joints of mice that did not undergo surgery (*n* = 6) and ACLT mice treated with lentiviral vectors expressing Luc (*n* = 8), YAP (*n* = 8), or FOXD1 (*n* = 8). (D) Representative images of P16 immunostaining in the joint cartilage of mice and the statistical analysis (no surgery, *n* = 6; ACLT-Luc, *n* = 8; ACLT-YAP, *n* = 8; ACLT-FOXD1, *n* = 8). Scale bar, 50 μm. Data are presented as the mean ± SD, **P < 0.01, ***P < 0.001. (E Left) Safranin O and Fast Green staining of articular cartilage from mice that did not undergo surgery and ACLT mice that were treated as indicated. Scale bar. 100 μm. (E Right), OARSI scores of articular joints (no surgery, *n* = 6; ACLT-Luc, *n* = 8; ACLT-YAP, *n* = 8; ACLT-FOXD1, *n* = 8). Data are presented as the mean ± SD, **P < 0.01, ***P < 0.001. (F) RNA-seq analysis of articular joints. The numerical data underlying this figure are included in S8 Data. ACLT, anterior cruciate ligament transection; Bmf, BCL2 modifying factor; Casp4, caspase 4, Chad, chondroadherin; Cidec, cell death-inducing DFFA-like effector c; Cilp, cartilage intermediate layer protein, nucleotide pyrophosphohydrolase; Cish, cytokine inducible SH2-containing protein; Col10a1, collagen, type X, alpha 1; Comp, cartilage oligomeric matrix protein; Cxcl9, chemokine (C-X-C motif) ligand 9; Dapk1, death associated protein kinase 1; FOXD1, forkhead box D1; Ihh, Indian hedgehog; Ifit3, interferon-induced protein with tetratricopeptide repeats 3; Irs2, insulin receptor substrate 2; Luc, luciferase; Matn3, matrilin 3; OARSI, Osteoarthritis Research Society International; Pawr, PRKC, apoptosis, WT1, regulator; Pdcd1, programmed cell death 1; RNA-seq, RNA sequencing; RT-qPCR, reverse transcription quantitative polymerase chain reaction; Ska2, spindle and kinetochore associated complex subunit 2; Tnc, tenascin C; Tnfrsf9, tumor necrosis factor receptor superfamily, member 9; YAP, Yes-associated protein.

The elimination of local senescent cells using pharmacological or genetic approaches have been proven effective in attenuating age-associated bone loss and development of post-traumatic osteoarthritis in rodents [51,53]. Given the ability of YAP or FOXD1 to rejuvenate senescent MSCs, we hypothesized that intra-articular injection of lentiviral vectors expressing YAP or FOXD1 might exert a therapeutic effect on osteoarthritis. To test this, we performed an anterior cruciate ligament transection (ACLT) surgery widely used to trigger osteoarthritis in mice and then administrated the lentiviruses expressing flag-tagged Luc, YAP, or FOXD1 intra-articularly (Fig 6A). The lentiviral vectors steadily expressed exogenous proteins in and around the joints receiving virus injection for at least 7 weeks (S8A Fig). High expression levels of YAP and FOXD1 were detectable by RT-qPCR (Fig 6B and 6C); immunohistochemical analysis of the flag-tagged Luc, YAP, and FOXD1 further verified the persistent infection of the lentiviruses and expression of indicated proteins primarily in the superficial zone of articular cartilage (S8B Fig). As expected, ACLT induced the accumulation of P16-positive senescent cells in the articular cartilage, particularly in the superficial zone of cartilage, of the osteoarthritis mice (Fig 6D), which was accompanied by decreased levels of YAP and FOXD1 (S8C and S8D Fig). YAP or FOXD1 gene therapy reduced the number of senescent cells and alleviated ACLT-induced articular cartilage erosion and clefts (Fig 6D and 6E). Consistently, a substantial proportion of gene expression changes in the joints induced by ACLT were reversed by YAP or FOXD1 gene therapy (S8C–S8F Fig and S7 Data). For instance, increased expression of genes associated with inflammation (*Mmp13*, *Il6*, etc.), cellular senescence (*P21*, *Serpine1*, etc.), and cell apoptosis (*Dapk1*, *Casp4*, etc.) were observed in ACLT-induced osteoarthritis joints, and the expression levels of most of these genes were diminished upon YAP or FOXD1 treatment. Moreover, the YAP or FOXD1 treatment enhanced the expression of proliferation markers (*Ki67*, *Aspm*, etc.) and chondrocyte differentiation-related genes (*Col2a1*, *Acan*, etc.) (Fig 6F). Taken together, these data suggest that the YAP- or FOXD1-mediated alleviation of cellular senescence in local bone joints helps create a prochondrogenic environment and alleviates disease symptoms.

## Discussion

Cellular senescence and stem cell exhaustion are hallmarks of aging [54]. Accelerated attrition of the MSC pool has been observed in human stem cell and mouse models of premature aging disorders, including WS and Hutchinson Gilford progeria syndrome (HGPS) [37,55]. Transplantation of mesoderm-derived stem cells from young animals increases the lifespan of progeroid mice [56]. Quercetin has been shown to alleviate MSC senescence [57], improve physical function, and increase lifespan in aged mice [58]. From this perspective, senescent MSCs could be good therapeutic targets for aging-associated degenerative disorders. In this study, we presented several lines of evidence supporting a geroprotective role of YAP and FOXD1 in rejuvenating hMSCs: (1) YAP is required for preventing premature senescence of hMSCs; (2) YAP transcriptionally activates *FOXD1* expression whereas YAP deficiency results in down-regulation of *FOXD1*, which contributes to the early-onset of cellular aging; and (3) lentiviral gene transfer of YAP or FOXD1 alleviates cellular senescence and osteoarthritis. Our findings define a critical role of the YAP–FOXD1 axis in regulating hMSC aging, which highlights new avenues for translation into geriatric and regenerative medicine.

The Hippo-YAP/TAZ signaling pathway is an evolutionarily conserved pathway that regulates cell proliferation and apoptosis. Here, we focused on the function of YAP and/or TAZ in regulating hMSC senescence. We generated isogenic YAP- or TAZ-deficient hMSCs. Compared with WT cells, *TAZ*^−/−^ hMSCs showed minimal effect in cell growth, whereas *YAP*^−/−^ hMSCs exhibited accelerated senescence. The cytoplasmic localization of TAZ underlays its inactivation in hMSCs, whereas nuclear YAP was essential for counteracting hMSC senescence. Consistent with our observations, emerging studies have revealed the differences between YAP and TAZ. For example, TAZ promotes the myogenic differentiation of myoblasts at late stages of myogenesis, whereas YAP inhibits this process in mice [59]. However, in-depth insights into the molecular mechanisms underlying these functional differences require further investigations.

*FOXD1* is a new downstream target of YAP, loss of which mediates the senescent phenotype of YAP-deficient hMSCs. As a member of the forkhead box family of transcription factors, FOXD1 is known to regulate kidney development during organogenesis [60,61]. Recently, FOXD1 has been shown to promote cell proliferation by targeting the sonic hedgehog pathway and cyclin-dependent kinase inhibitors [62,63]. FOXD1 also facilitates the reprogramming of mouse embryonic fibroblasts (MEFs) into induced pluripotent stem cells (iPSCs) [64]. Here, we identified a geroprotective role for FOXD1 as a transcriptional target of YAP in rejuvenating hMSCs. Overexpression of YAP or FOXD1 delayed replicative and pathological senescence, implying a therapeutic potential of targeting the YAP–FOXD1 axis to relieve aging-associated degenerative diseases.

In a therapeutic context, we provided a proof-of-concept evidence that intra-articular lentiviral transduction of a single protein exerted therapeutic effects on ACLT-induced osteoarthritis, an age-related disorder. Because ACLT-induced osteoarthritis is accompanied by the accumulation of senescent cells [65], efforts have been made on chemical-induced elimination of senescent cells to alleviate osteoarthritis in mouse models [50,51]. With gene therapy offering novel therapeutic options for osteoarthritis [66], intra-articular injection presents a minimally invasive procedure that avoids conventional barriers to joint entry, increases bioavailability, and lowers systemic toxicity [67]. For the first time, our study shows that the intra-articular injection of lentiviruses expressing YAP or FOXD1 reduces the number of senescent cells, inhibits articular inflammation and cartilage erosion, and ameliorates the pathological symptoms. Therefore, gene therapy via the introduction of geroprotective factors aiming at rejuvenating senescent cells may represent a new avenue to treating osteoarthritis in the future.

## Materials and methods

### Ethics statement

All animal experiments were conducted in compliance with animal protocols approved by the Chinese Academy of Science Institutional Animal Care and Use Committee, licensed by the Science and Technology Commission of Beijing Municipality (SYXK-2016-0026). All mice were housed under a 12-hour light/dark cycle at constant temperature (22°C). Food and water were available ad libitum. Mice were anaesthetized using isoflurane and euthanized with CO_2_ followed by cervical dislocation.

### Cell culture

Human H9 ESCs as well as derived *YAP*^−/−^ and *TAZ*^−/−^ hESCs were maintained on feeder layers of mitomycin C–inactivated MEFs in hESC medium [68] (DMEM/F12 [Thermo Fisher Scientific, Waltham, MA], 20% Knockout Serum Replacement [Thermo Fisher Scientific], 0.1 mM nonessential amino acids [NEAAs; Thermo Fisher Scientific], 2 mM GlutaMAX [Thermo Fisher Scientific], 1% penicillin/streptomycin [Thermo Fisher Scientific], 55 μM β-mercaptoethanol [Thermo Fisher Scientific], and 10 ng/ml bFGF [Joint Protein Central, Incheon, Korea]) or on Matrigel (BD Biosciences, San Jose, CA, USA) in mTeSR medium (STEMCELL Technologies, Vancouver, Canada). hESCs derived hMSCs and BM-hMSCs (purchased from Lonza, Basel, Switzerland) were cultured in hMSC medium (αMEM + GlutaMAX [Thermo Fisher Scientific], 10% fetal bovine serum [Gibco, Cat: 10099–141, Lot: 1616964], 1% penicillin/streptomycin [Thermo Fisher Scientific], and 1 ng/ml bFGF [Joint Protein Central]). No mycoplasma contamination was observed during cell culture.

### Antibodies

Antibodies used for western blotting, immunostaining and flow cytometry included anti-YAP (15407; Santa Cruz Biotechnology, Santa Cruz, CA), anti-YAP (52771; Abcam, Cambridge, MA), anti-TAZ (4883; Cell Signaling Technology, Danvers, MA), anti-GAPDH (25778; Santa Cruz Biotechnology), anti-P16 (550834; BD), anti-P21 (2947s; Cell Signaling Technology), anti-P53 (1101; Abcam), anti-β-tubulin (5274; Santa Cruz Biotechnology), anti-β-actin (69879; Santa Cruz Biotechnology), anti-Pan TEAD (13295; Cell Signaling Technology), anti-FOXD1 (PA5-27142; Thermo Fisher Scientific), anti-NANOG (21624; Abcam), anti-OCT3/4 (5279; Santa Cruz Biotechnology), anti-SOX2 (17320; Santa Cruz Biotechnology), anti-TUJ1 (T2200; Sigma, St. Louis, MO), anti-FOXA2 (8186; Cell Signaling Technology), anti-SMA (A5228; Sigma), anti-Ki67 (VP-RM04; Vector Labs, Burlingame, CA), anti-LAP2 (611000; BD), anti-HP1α (2616S; Cell Signaling Technology), anti-HP1γ (2619; Cell Signaling Technology), anti-Aggrecan (AF1220; R&D, Minneapolis, MN), anti-Osteocalcin (MAB1419; R&D), anti-FABP4 (AF3150; R&D), anti-CD73 (550741; BD Bioscience), anti-CD90 (555595; BD Bioscience), anti-CD105 (17–1057; eBioscience, San Diego, CA), anti-CD29 (303004; Biolegend, San Diego, CA), anti-CD44 (550989; BD Bioscience), anti-CD13 (301705; Biolegend), anti-CD166 (343903; Biolegend), anti-HLA-ABC (560168; BD Bioscience), anti-CD34 (555822; BD Biosciences), anti-CD43 (580198; BD Biosciences), anti-CD45 (555482; BD Biosciences), anti-CD14 (555398; BD Biosciences), anti-CD19 (555415; BD Biosciences), anti-PDPN (17-9381-41; eBioscience), and anti-CD164 (324805; Biolegend).

### Generation of *YAP*^−/−^ and *TAZ*^−/−^ hESCs

CRISPR/Cas9-mediated gene targeting was performed using previously described methods, with some modifications [69]. The *YAP* or *TAZ* gRNA was cloned into the gRNA vector (Addgene #41824). The donor plasmid for homologous recombination containing homology arms and a neo cassette was described previously [70]. Briefly, 5 × 10^6^ H9 ESCs were mixed with the plasmid cocktail and electroporated. After electroporation, cells were plated on a G418-resistant MEF feeder layer. Two days later, cells were treated with 100 μg/ml G418 (Gibco, 10131027) for screening. After 2 weeks of selection, G418-resistant clones were manually picked, transferred to 96-well plates, and expanded for genotyping. Gene-targeted clones were identified using genomic PCR. gRNA sequences and primers are listed in S1 Data.

### Directed differentiation of hESCs into hMSCs

hMSCs were differentiated from hESCs as previously described [70–72]. Briefly, hESCs were dissociated into embryoid bodies and then plated on Matrigel-coated plates in differentiation medium (α-MEM + GlutaMAX [Thermo Fisher Scientific], 10% FBS [Gibco, Cat: 10099–141, Lot: 1616964], 1% penicillin/streptomycin [Thermo Fisher Scientific], 10 ng/ml bFGF, and 5 ng/ml TGFβ [HumanZyme, Chicago, IL]). After 10 days, the confluent MSC-like cells were passaged to gelatin-coated plates and sorted by FACS to purify CD73/CD90/CD105 triple-positive hMSCs, which were further characterized by flow cytometry analysis of the surface antigens, including CD166, CD29, CD44, CD13, HLA-ABC, CD34, CD43, CD45, CD14, CD19, PDPN, and CD164. The functionality of hMSCs was verified by differentiation to osteoblasts, chondrocytes, and adipocytes.

### Lentiviral CRISPR/Cas9-mediated *YAP* or *FOXD1* KO

Lentiviral CRISPR/Cas9-mediated gene editing was performed as previously described [42]. Briefly, the sgRNA targeting *YAP* or *FOXD1* was cloned into lentiCRISPRv2 vector (Addgene #52961), which contains 2 expression cassettes, hSpCas9 and the chimeric sgRNA. Then, the plasmids were packaged into lentiviruses and transduced into hMSCs; 72 hours later, transduced cells were treated with 1 μg/ml puromycin (Gibco, A1113803) for enriching transduced cells. The sgRNA sequences are listed in S1 Data.

### Generation of TEADs KD/KO hMSCs

We generated 2 lentiviral constructs to silence the expression of TEAD1, 2, 3, and 4: one containing an shRNA targeting *TEAD1*, *TEAD3*, and *TEAD4* and the other containing an sgRNA targeting *TEAD2* [73]. The shRNA was cloned into the PLVTHM vector (Addgene #12247), and the sgRNA was cloned into lentiCRISPRv2. We then cotransduced these lentiviral constructs into hMSCs. Seventy-two hours later, transduced cells were enriched by treatment with 1 μg/ml puromycin (Gibco, A1113803). The targeting sequences are listed in S1 Data.

### Lentivirus packaging

For packaging of the lentivirus, HEK293T cells were cotransfected with lentiviral vectors, psPAX2 (Addgene #12260) and pMD2.G (Addgene #12259). Viral particles were collected by ultracentrifugation at 19,400 g for 2.5 hours.

### Flow cytometry analysis

For the cell cycle analysis, the Click-iT EdU Alexa Fluor 647 Flow Cytometry Assay Kit (C-10419; Molecular Probes, Eugene, OR) was used according to the manufacturer’s instructions. For ROS measurements, living cells were incubated with ROS indicators (1 μM CM-H2DCFDA, C6827; Molecular Probes). All experiments were measured with an LSRFortessa cell analyzer (BD), and data were analyzed using FlowJo software (TreeStar, Ashland, OR).

### Immunofluorescence staining

Cells were fixed with 4% paraformaldehyde for 30 minutes, washed with PBS, permeabilized with 0.4% Trion X-100 in PBS, and then blocked with 10% donkey serum (Jackson ImmunoResearch Labs, West Grove, PA). Afterwards, cells were incubated with primary antibodies in blocking solution at 4°C overnight, followed by an incubation with the corresponding secondary antibodies and Hoechst 33342 for 1 hour at room temperature.

### SA-β-gal staining

The SA-β-gal staining of hMSCs was conducted using a previously described method [74].

### Protein, RNA, and DNA analyses

For western blotting, cells were lysed in RIPA buffer containing a protease inhibitor cocktail (Roche) and quantified with a BCA kit. Generally, 20 μg of cell lysate was subjected to SDS-PAGE and electrotransferred to a PVDF membrane (Millipore, Billerica, MA). Then, the membrane was incubated with primary and HRP-conjugated secondary antibodies. Western blot data were quantified with Image Lab software for the ChemiDoc XRS system (Bio-Rad, Hercules, CA). For RT-qPCR, cellular total RNA was extracted using TRIzol (Thermo Fisher Scientific), and genomic DNA was removed with a DNA-free Kit (Ambion, Austin, TX), followed by cDNA synthesis with the GoScript Reverse Transcription System (Promega, Madison, WI). RT-qPCR was performed with qPCR Mix (TOYOBO, Tokyo, Japan) in a CFX384 Real-Time system (Bio-Rad). For genomic PCR, genomic DNA was extracted with a DNA extraction kit (TIANGEN, Beijing, China), and PCR was conducted using PrimeSTAR (TAKARA, Tokyo, Japan).

### Clonal expansion assay

Two thousand cells were seeded in each well of a 12-well plate and then cultured until clear cell colonies formed to determine the clonal expansion abilities of hMSCs. The relative colony area was then determined by performing crystal violet staining and measured using ImageJ software.

### Dual Luc assay

The indicated fragments of the *FOXD1* promoter were amplified by PCR and cloned into the pGL3-Basic vector (Promega). The mutant of pGL3-*FOXD1* promoter 2-Luc was constructed with the Fast Mutagenesis System kit (FM111; Transgen Biotech, Beijing, China). PGL3-*FOXD1* promoter 2-Luc or PGL3-*FOXD1* promoter 2(mut)-Luc was transfected into hMSCs together with vectors expressing the proteins of interest and Renilla-Luc, which was used to normalize the transfection efficiency. For detection of the 8 × GTIIC-Luc activity, the 8 × GTIIC reporter (Addgene #34615) and Renilla-Luc plasmids were cotransfected into hMSCs. Cells were harvested 72 hours later using the Dual-Luciferase Reporter Assay System (Vigorous Biotechnology, Beijing, China) and assayed according to the manufacturer’s instructions.

### ChIP

ChIP was performed using a previously reported protocol with minor modifications [75]. Briefly, cells were cross-linked with 1% (v/v) formaldehyde for 15 minutes at room temperature, and the reaction was terminated by the addition of 125 mM glycine and an incubation for 5 minutes at room temperature. Then, cells were scraped and lysed in lysis buffer. After sonication, protein-DNA complexes were incubated with antibody-coupled Protein A beads at 4°C overnight. After elution and reverse cross-linking at 68°C, DNA was purified by phenol/chloroform extraction and ethanol precipitation and then subjected to qPCR analysis. Antibodies for ChIP included anti-YAP (14074; Cell Signaling Technology), anti-TAZ (4883; Cell Signaling Technology), anti-TEAD4 (101184; Santa Cruz Biotechnology), and normal rabbit IgG (2027; Santa Cruz Biotechnology) as a negative control.

### Animal experiments

For the teratoma analysis, 5 × 10^6^ hESCs were subcutaneously injected into NOD-SCID mice (6 to 8 weeks, male). After 8 to 12 weeks, the tumors were excised, fixed, dehydrated, embedded in O.C.T. compound, sectioned while frozen, and analyzed by immunostaining.

For hMSC transplantation assays, 1 × 10^6^ hMSCs transduced with a lentivirus expressing Luc were injected into the midportion of the TA muscle of nude mice (6 to 8 weeks, male). Then, 0, 1, 3, 5, and 7 days after transplantation, mice were treated with D-luciferin (GoldBio, St. Louis, MO) and imaged with an IVIS spectrum imaging system (XENOGEN, Caliper, Waltham, MA). Bioluminescence images were acquired in “auto” mode.

For RS-hMSC–induced osteoarthritis, we transplanted PBS, young hMSCs, RS hMSCs, and RS hMSCs overexpressing YAP or FOXD1 intra-articularly into the joints of NOD-SCID mice (6 to 8 weeks, male). Firstly, the mice were anaesthetized using isoflurane, and skin around the joints were shaved. For each injection, the needle was inserted beneath the middle patellar ligament, and a volume of 10 μl containing either PBS or 3 × 10^6^ cells was injected intra-articularly. One month later, the mice were euthanized, and the joints were collected for mRNA quantification and histological assessments.

For surgically induced osteoarthritis, we performed ACLT surgery on 8-week-old male C57BL/6 mice. Animals were anaesthetized, and their hindlimbs were shaved. After the opening of the joint capsule, the anterior cruciate ligament was transected with microscissors under a surgical microscope. After irrigation with saline to remove tissue debris, the skin incision was closed. Then, 7 days later, a total volume of 10 μl of the indicated lentivirus was injected intra-articularly. At week 8, the mice were euthanized, and the joints were collected for mRNA quantification and histological assessments.

### Histology

Mouse joints were fixed with 4% paraformaldehyde overnight, decalcified with 5% methanoic acid for 7 days, and embedded in paraffin. Sections (5 μm) were cut from the paraffin blocks and stained with Fast Green FCF (0.02%) and Safranin O (0.1%). Joint pathology was quantified using the OARSI scoring system [13].

### Immunochemistry

For immunohistochemical staining, paraffin-embedded tissue sections were subjected to a heat-mediated antigen retrieval procedure, and then endogenous peroxidases were blocked with hydrogen peroxide. Next, tissue sections were incubated with a primary antibody overnight. Finally, the appropriate secondary antibody (ZSGB-BIO, Beijing, China) was added to the sections, which were then incubated for 30 minutes. Antigen-positive cells were visualized using the DAB Substrate kit (ZSGB-BIO). Anti-P16 antibody (54210; Abcam) and anti-flag (166355; Santa Cruz Biotechnology) were used as the primary antibodies.

### CNV identification

First, genomic DNA was extracted using the DNeasy Blood and Tissue Kit (Qiagen, Duesseldorf, Germany) according to the manufacturer’s instructions. DNA was sheared into fragments of approximately 300 bp using Covaris, and then the library of the fragmented DNA was constructed using the NEBNext ultra DNA Library Prep Kit for Illumina (NEB, Beverly, MA), according to the manufacturer’s protocol. The libraries were sequenced on an Illumina HiSeq 4000 platform. For CNV identification, we used the published R package HMMcopy [76]. Briefly, the genome was binned into consecutive 1 Mb windows with read Counter, and then we calculated the absolute number of reads detected in each window. We estimated the copy number with GC and mappability corrections with HMMcopy.

### RNA-seq library preparation and sequencing

Total RNA was extracted from cultured human cells or mouse joints using the RNeasy Mini Kit (Qiagen) according to the manufacturer’s protocol. For cells, 1 × 10^6^ cells were analyzed in biological triplicate. For mouse joints, we mixed the RNA extracted from the sample group, and then divided the sample into 3 technical replicates. One to two micrograms of total RNA was used to construct sequencing libraries using the NEBNext Ultra RNA Library Prep Kit for Illumina (NEB). The libraries were sequenced on an Illumina HiSeq 4000 platform. RNA-seq reads were aligned to the hg19 or mm10 reference genome using TopHat2 software [77]. The analysis of differentially expressed genes was performed using DESeq2 [78] based on read counts.

### TEAD-binding site analysis

Promoter for TEAD-binding sites analysis was defined as 3 kb upstream and 500 bp downstream of TSS. TEAD-binding sites with *P* < 1.0 × 10^−4^ among the promoter regions were found by FIMO (http://meme-suite.org/doc/fimo.html) using the TEAD motif downloaded from JASPAR database (http://jaspardev.genereg.net/).

### Statistical analysis

Results are presented as the mean ± SD. Two-tailed Student *t* tests were used to compare differences between treatments. *P* < 0.05, *P* < 0.01, and *P* < 0.001 were considered statistically significant ("*", "**", and "***", respectively).

## Supporting information

S1 Fig Generation and characterization of *YAP*^−/−^ and *TAZ*^−/−^ hESCs. (A) Schematic showing the KO of *YAP* in hESCs using CRISPR/Cas9-mediated gene targeting. Exon 1 was removed from *YAP*. (B) Schematic showing the KO of *TAZ* in hESCs using CRISPR/Cas9-mediated gene targeting. Exon 2 was removed from *TAZ*. (C) Genomic PCR and RT-qPCR analyses showing the *YAP* deletion. Data are presented as the mean ± SD, *n* = 3, ***P < 0.001. (D) Genomic PCR and RT-qPCR analyses showing the *TAZ* deletion. Data are presented as the mean ± SD, *n* = 3, ***P < 0.001. (E) Immunostaining for representative markers of the 3 germ layers in teratomas that were developed from WT, *YAP*^−/−^, and *TAZ*^−/−^ hESCs. Scale bar, 75 μm. (F) G-banded karyotyping analysis of WT, *YAP*^−/−^, and *TAZ*^−/−^ hESCs showing normal karyotypes. (G) Summary of the generated total and *YAP*/*TAZ* dKO clone numbers. The numerical data underlying this figure are included in S8 Data. Cas9, CRISPR associated protein 9 nuclease; CRISPR, Clustered Regularly Interspaced Short Palindromic Repeats; Ctrl, control; dKO, double knockout; ESC, embryonic stem cell; FOXA2, forkhead box A2; hESC, human embryonic stem cell; HR, homologous recombination; KO, knockout; RT-qPCR, reverse transcription quantitative polymerase chain reaction; SMA, smooth muscle actin; TAZ, transcriptional coactivator with PDZ-binding motif; TUJ1, beta-tubulin III; WT, wild type; YAP, Yes-associated protein.
(TIF)

S2 Fig Characterization of WT, *YAP*^−/−^, and *TAZ*^−/−^ hMSCs. (A) Surface antigen expression levels of WT, *YAP*^−/−^, and *TAZ*^−/−^ hMSCs. Data are presented as the mean ± SD, *n* = 3. (B) Characterization of the multilineage differentiation potential of WT, *YAP*^−/−^, and *TAZ*^−/−^ hMSCs. Toluidine blue and Aggrecan staining were used to evaluate chondrogenesis. Von Kossa and Osteocalcin staining were used to evaluate osteogenesis. Oil Red O and FABP4 staining were used to evaluate adipogenesis. Scale bar, 25 μm. (C) The diameters of chondrocyte spheres were measured. Data are presented as the mean ± SD, *n* = 10, *P < 0.05, **P < 0.01. (D) Areas of Von Kossa–positive cells were calculated. Data are presented as the mean ± SD, *n* = 3, **P < 0.01. (E) Areas of Oil Red O-positive cells were calculated. Data are presented as the mean ± SD, *n* = 3. (F) Representative images of immunofluorescence staining for HP1α, HP1γ, LAP2, and Ki67 in hMSCs. Scale bar, 50 μm. (G) Quantification of HP1α−, HP1γ−, LAP2−, and Ki67-positive nuclei in WT, *YAP*^−/−^, and *TAZ*^−/−^ hMSCs. More than 100 randomly selected nuclei were analyzed from each group. Data are presented as the mean ± SD, **P < 0.01. The numerical data underlying this figure are included in S8 Data. hMSC, human mesenchymal stem cell; HP1α, heterochromatin protein 1 alpha; HP1γ, heterochromatin protein 1 gamma; LAP2, lamina-associated protein 2; ns, not significant; PDPN, podoplanin; TAZ, transcriptional coactivator with PDZ-binding motif; WT, wild type; YAP, Yes-associated protein.
(TIF)

S3 Fig YAP deficiency in hMSCs accelerates cellular senescence. (A) Flow cytometry analysis of cellular ROS levels using H2DCFDA probes. (B) WT and *TAZ*^−/−^ hMSCs were transduced with the lentivirus expressing Luc and injected into the TA muscle of immunodeficient mice. Luc activities were imaged at day 0, 1, 3, 5, and 7 after cell implantation. The representative images at day 0 and day 7 are shown. (C) The Luc activities were presented as the ratios of *TAZ*^−/−^ to WT cells (log_10_ (fold)), mean ± SD, *n* = 5. (D) Western blot analysis of YAP in hMSCs transduced with lentiviruses expressing NTC or *YAP* sgRNA, as well as CRISPR/Cas9. GAPDH was used as a loading control (left). The protein levels normalized with GAPDH were shown as fold change relative to lenti-NTC–sgRNA–transduced hMSCs. Data are presented as the mean ± SD, *n* = 3, ***P < 0.001 (right). (E) SA-β-gal analysis of hMSCs transduced with lentiviruses expressing NTC or *YAP* sgRNA, as well as CRISPR/Cas9. Data are presented as the mean ± SD, *n* = 3, ***P < 0.001. (F) Cell growth curves of *YAP*^−/−^ hMSCs transduced with a lentiviral vector encoding Luc or YAP. Data are presented as the mean ± SD, *n* = 3, **P < 0.01. (G) Analysis of the clonal expansion of *YAP*^−/−^ hMSCs lentivirally expressing Luc or YAP. Areas of crystal violet–positive cells were calculated using ImageJ software. Data are presented as the mean ± SD, *n* = 3, ***P < 0.001. (H) Western blot analysis showing decreased expression of P16 and P21 upon the ectopic expression of YAP in *YAP*^−/−^ hMSCs. β-tubulin was used as a loading control (left). The protein levels normalized with β-tubulin were shown as fold change relative to WT hMSCs. Data are presented as the mean ± SD, *n* = 3, *P < 0.05, **P < 0.01. (I) ROS detection in WT hMSCs transduced with the lentivirus expressing Luc and *YAP*^−/−^ hMSCs transduced with lentiviruses expressing Luc or YAP. (J) *YAP*^−/−^ hMSCs overexpressing GFP plus Luc and *YAP*^−/−^ hMSCs overexpressing YAP plus Luc were implanted into the TA muscles of immunodeficient mice. Luc activities were imaged at day 0, 1, 3, 3, 5, and 7 after cell implantation. Representative images at day 0 and day 7 are shown. The numerical data underlying this figure are included in S8 Data. Cas9, CRISPR associated protein 9 nuclease; CRISPR, Clustered Regularly Interspaced Short Palindromic Repeats; GAPDH, glyceraldehyde-3-phosphate dehydrogenase; GFP, green fluorescent protein; H2DCFDA, 2′,7′-dichlorodihydrofluorescein diacetate; hMSC, human mesenchymal stem cell; lenti, lentivirus; Luc, luciferase; MSC, mesenchymal stem cell; NTC, non-targeting control; ns, not significant; ROS, reactive oxygen species; SA-β-gal, senescence-associated-β-galactosidase; sgRNA, single guide RNA; TA, tibialis anterior; TAZ, transcriptional coactivator with PDZ-binding motif; WT, wild type; YAP, Yes-associated protein.
(TIF)

S4 Fig YAP and TEAD, but not TAZ, activates *FOXD1* transcription. (A) Clonal expansion analysis of Ctrl and TEADs KD/KO hMSCs. Areas of crystal violet–positive cells were calculated using ImageJ software. Data are presented as the mean ± SD, *n* = 3, ***P < 0.001. (B) Western blots for P16 and P21 in Ctrl and TEADs KD/KO hMSCs. GAPDH was used as a loading control (left). The protein levels normalized with GAPDH were shown as fold change relative to Ctrl hMSCs. Data are presented as the mean ± SD, *n* = 3, *P < 0.05, **P < 0.01. (C) Pearson correlation coefficients for gene expression in WT, *YAP*^−/−^, and *TAZ*^−/−^ hMSCs. (D) Venn diagrams showing DEGs in *YAP*^−/−^ and *TAZ*^−/−^ hMSCs relative to WT hMSCs. (E) The top 5 GO BPs enriched among the DEGs in *YAP*^−/−^ hMSCs. (F) ChIP-qPCR for TAZ enrichment within different *FOXD1* pro regions (Pro 1 and Pro 2) containing putative TEAD binding motifs. Data are presented as the mean ± SD, *n* = 3. (G) The *FOXD1* pro containing the Pro 2 region and a mutation were cloned upstream of a Luc reporter, and the Luc activities were measured after transfection of GFP or TAZ. Data are presented as the mean ± SD, *n* = 3. The numerical data underlying this figure are included in S8 Data. BP, biological process; ChIP-qPCR, chromatin immunoprecipitation quantitative polymerase chain reaction; Ctrl, control; DEG, differentially expressed gene; FOXD1, forkhead box D1; GAPDH, glyceraldehyde-3-phosphate dehydrogenase; GFP, green fluorescent protein; GO, gene ontology; hMSC, human mesenchymal stem cell; KD, knockdown; KO, knockout; mut, mutant; ns, not significant; pro, promoter; TAZ, transcriptional coactivator with PDZ-binding motif; TEAD, TEA domain transcriptional factor; WT, wild type; YAP, Yes-associated protein.
(TIF)

S5 Fig FOXD1 KO induces hMSC senescence. (A) Genomic sequencing of the *FOXD1* locus in NTC and FOXD1 KO hMSCs. (B) Clonal expansion analysis of NTC and FOXD1 KO hMSCs. Areas of crystal violet–positive cells were calculated using ImageJ software. Data are presented as the mean ± SD, *n* = 3, **P < 0.01. (C) Western blot analysis for P16 and P21 in NTC and FOXD1 KO hMSCs. GAPDH was used as a loading control (left). The protein levels normalized with GAPDH were shown as fold change relative to NTC hMSCs. Data are presented as the mean ± SD, *n* = 3, *P < 0.05. (D) PC analysis of WT, *YAP*^−/−^, NTC, and FOXD1 KO hMSCs. (E) Comparison of the GSEA of cell cycle and cell adhesion genes between WT and *YAP*^−/−^ hMSCs and between NTC and FOXD1 KO hMSCs. (F) SA-β-gal analysis of TEADs KD/KO hMSCs transduced with lentiviruses expressing Luc or YAP. Scale bar, 100 μm. Data are presented as the mean ± SD, *n* = 3. (G) SA-β-gal analysis of FOXD1 KO hMSCs transduced with lentiviruses expressing Luc or YAP. Scale bar, 100 μm. Data are presented as the mean ± SD, *n* = 3. The numerical data underlying this figure are included in S8 Data. FOXD1, forkhead box D1; GAPDH, glyceraldehyde-3-phosphate dehydrogenase; GSEA, Gene Set Enrichment Analysis; hMSC, human mesenchymal stem cell; Indel, insertion and deletion; KD, knockdown; KO, knockout; Luc, luciferase; ns, not significant; NTC, non-targeting control; PAM, protospacer-adjacent motif; PC, principal component; SA-β-gal, senescence-associated-β-galactosidase; TEAD, TEA domain transcriptional factor; WT, wild type; YAP, Yes-associated protein.
(TIF)

S6 Fig YAP or FOXD1 expression delays replicative and premature cellular senescence. (A) Growth curve of RS hMSCs transduced with lentiviruses expressing Luc, YAP, or FOXD1. (B) Cell cycle analysis of RS hMSCs transduced with lentiviruses expressing Luc, YAP, or FOXD1. Data are presented as the mean ± SD, *n* = 3, **P < 0.01, ***P < 0.001. (C) Clonal expansion abilities of RS hMSCs transduced with lentiviruses expressing Luc, YAP, or FOXD1. Data are presented as the mean ± SD, *n* = 3, ***P < 0.001. (D) Growth curves of WS hMSCs transduced with lentiviruses expressing Luc, YAP, or FOXD1. (E) RT-qPCR of aging-associated markers in WS hMSCs transduced with lentiviruses expressing Luc, YAP, or FOXD1. Data are presented as the mean ± SD, *n* = 3, *P < 0.05, **P < 0.01, ***P < 0.001. (F) Measurement of Luc activity using IVIS at day 0 and day 7 after in vivo implantation of WS hMSCs transduced with lentiviruses expressing YAP or GFP. Data are presented as the ratios of YAP to GFP (log_10_ (fold)), mean ± SD, *n* = 3, ***P < 0.001. (G) Measurement of Luc activity using IVIS at day 0 and day 7 after in vivo implantation of WS hMSCs transduced with lentiviruses expressing FOXD1 or GFP. Data are presented as the ratios of FOXD1 to GFP (log_10_ (fold)), mean ± SD, *n* = 3, ***P < 0.001. The numerical data underlying this figure are included in S8 Data. FOXD1, forkhead box D1; GFP, green fluorescent protein; hMSC, human mesenchymal stem cell; IVIS, in vivo imaging system; LAP2, lamina-associated protein 2; LMNB1, Lamin B1; Luc, luciferase; ns, not significant; RS, replicative-senescent; RT-qPCR, reverse transcription quantitative polymerase chain reaction; WS, Werner syndrome; YAP, Yes-associated protein.
(TIF)

S7 Fig Implanted senescent hMSCs induces osteoarthritis in mice. (A) Schematic of the time course for experiments in B–D. (B) Safranin O and Fast Green staining of articular cartilage from mice that transplanted with indicated hMSCs. Representative images are shown. Scale bar, 100 μm. (C) OARSI scores of articular joints (PBS, *n* = 5; young hMSCs, *n* = 5; RS hMSCs, *n* = 6; RS hMSCs overexpressing YAP, *n* = 5; RS hMSCs overexpressing FOXD1, *n* = 5). Data are presented as the mean ± SD, *P < 0.05. (D) Heat map showing RT-qPCR analysis of the indicated genes associated with senescence, inflammation and chondrogenesis in joints transplanted with indicated hMSCs. Expression levels of the indicated genes were normalized to PBS group. The numerical data underlying this figure are included in S8 Data. Acan, aggrecan; Col2a1, collagen, type II, alpha 1; FOXD1, forkhead box D1; hMSC, human mesenchymal stem cell; Il1β, interleukin 1 beta; Mmp13, matrix metallopeptidase 13; ns, not significant; OARSI, Osteoarthritis Research Society International; RS, replicative-senescent; RT-qPCR, reverse transcription quantitative polymerase chain reaction; YAP, Yes-associated protein.
(TIF)

S8 Fig YAP or FOXD1 gene therapy attenuates the development of osteoarthritis. (A) Detection of the luminescence in the joints at different time points after injection of the lentivirus expressing Luc intra-articularly. The representative images are shown. (B) Immunohistochemical staining of indicated flag-tagged proteins in the joint cartilage of mice that did not receive surgery and of ACLT mice treated with lentiviruses expressing flag-tagged Luc, YAP, or FOXD1. Scale bar, 50 μm. (C and D) RT-qPCR analysis of YAP and FOXD1 expression in the joints of mice that did not undergo surgery (*n* = 6) and ACLT (*n* = 8). Data are presented as the mean ± SD, **P < 0.01, ***P < 0.001. (E and F) Heat maps showing relative mRNA expression levels of the differentially expressed genes in the ACLT-Luc group compared to the joints that did not undergo surgery. Genes were sorted by the fold change and *P* value (fold change > 2 or < 0.5, *P* < 0.01). Corresponding gene expression profiles obtained from ACLT-YAP and ACLT-FOXD1 groups are also shown. (G and H) Venn diagrams showing differentially expressed genes (no surgery versus ACLT-Luc; ACLT-YAP versus ACLT-Luc; ACLT-FOXD1 versus ACLT-Luc). The numerical data underlying this figure are included in S8 Data. ACLT, anterior cruciate ligament transection; FOXD1, forkhead box D1; Luc, luciferase; RT-qPCR, reverse transcription quantitative polymerase chain reaction; YAP, Yes-associated protein.
(TIF)

S1 Data Primer list. (XLSX)

S2 Data Differentially expressed genes in *YAP*^−/−^ and *TAZ*^−/−^ hMSCs, compared to WT hMSCs. hMSC, human mesenchymal stem cell; TAZ, transcriptional coactivator with PDZ-binding motif; WT, wild type; YAP, Yes-associated protein.
(XLSX)

S3 Data List of the most significant GO Biological Process terms associated with the differentially expressed genes in *YAP*^−/−^ hMSCs. GO, gene ontology; hMSC, human mesenchymal stem cell; YAP, Yes-associated protein.
(XLSX)

S4 Data Potential TEAD targets among the down-regulated genes in *YAP*^−/−^ hMSCs. hMSC, human mesenchymal stem cell; TEAD, TEA domain transcriptional factor; YAP, Yes-associated protein.
(XLSX)

S5 Data Differentially expressed genes identified by RNA-seq in FOXD1-sgRNA hMSCs compared to NTC-sgRNA hMSCs. FOXD1, forkhead box D1; hMSC, human mesenchymal stem cell; NTC, non-targeting control; RNA-seq, RNA sequencing; sgRNA, single guide RNA.
(XLSX)

S6 Data List of the most significant GO Biological Process terms associated with the differentially expressed genes in FOXD1-sgRNA hMSCs. FOXD1, forkhead box D1; GO, gene ontology; hMSC, human mesenchymal stem cell; sgRNA, single guide RNA.
(XLSX)

S7 Data Differentially expressed genes in ACLT joints transduced with lentiviruses expressing Luc, YAP, or FOXD1. ACLT, anterior cruciate ligament transection; FOXD1, forkhead box D1; Luc, luciferase; YAP, Yes-associated protein.
(XLSX)

S8 Data Numerical values of presented diagrams. (XLSX)

## Abbreviations

ACLT: anterior cruciate ligament transection
APC: allophycocyanin
BM-hMSC: human mesenchymal stem cells isolated from human bone marrow
CD: cluster of differentiation
ChIP: chromatin immunoprecipitation
Cas9: CRISPR associated protein 9 nuclease
CRISPR: Clustered Regularly Interspaced Short Palindromic Repeats
CNV: copy number variation
FITC: fluorescein isothiocyanate
FOXD1: forkhead box D1
G: gap
GO: gene ontology
hESC: human embryonic stem cell
hMSC: human mesenchymal stem cell
HLA-ABC: human leukocyte antigens, A, B and C
HP1α: heterochromatin protein 1 alpha
HP1γ: heterochromatin protein 1 gamma
HGPS: Hutchinson Gilford progeria syndrome
iPSC: induced pluripotent stem cell
KO: knockout
KD: knockdown
LAP2: lamina-associated protein 2
Luc: luciferase
M: mitosis
MEF: mouse embryonic fibroblast
MSC: mesenchymal stem cell
mut: mutant
NEAA: nonessential amino acid
NTC: non-targeting control
OARSI: Osteoarthritis Research Society International
PE: phycoerythrin
PDPN: podoplanin
RNA-seq: RNA sequencing
ROS: reactive oxygen species
RS: replicative-senescent
RT-qPCR: reverse transcription quantitative polymerase chain reaction
S: synthesis
SA-β-gal: senescence-associated-β-galactosidase
sgRNA: single guide RNA
TA: tibialis anterior
TAZ: transcriptional coactivator with PDZ-binding motif
TEAD: TEA domain transcriptional factor
TSS: transcriptional start site
WS: Werner syndrome
WT: wild type
YAP: Yes-associated protein
