# Supplementary figures and images for: Up-regulation of FOXD1 by YAP alleviates senescence and osteoarthritis
Source: PLoS Biol. 2019 Apr 1;17(4):e3000201. doi: 10.1371/journal.pbio.3000201 (PMC6459557; doi:10.1371/journal.pbio.3000201)

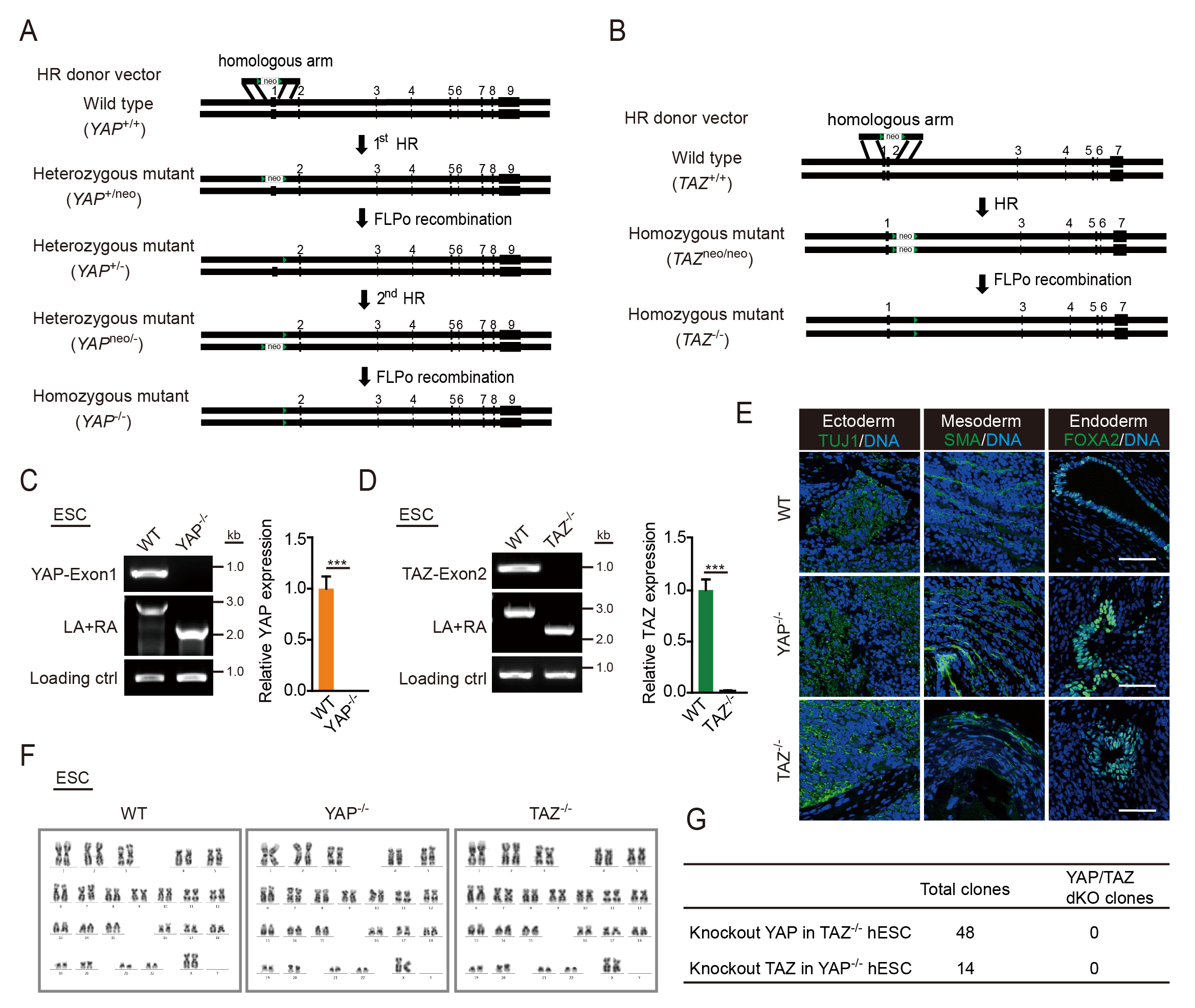

Supplement: S1 Fig — (A) Schematic showing the KO of YAP in hESCs using CRISPR/Cas9-mediated gene targeting. Exon 1 was removed from YAP. (B) Schematic showing the KO of TAZ in hESCs using CRISPR/Cas9-mediated gene targeting. Exon 2 was removed from TAZ. (C) Genomic PCR and RT-qPCR analyses showing the YAP deletion. Data are presented as the mean ± SD, n = 3, ***P < 0.001. (D) Genomic PCR and RT-qPCR analyses showing the TAZ deletion. Data are presented as the mean ± SD, n = 3, ***P < 0.001. (E) Immunostaining for representative markers of the 3 germ layers in teratomas that were developed from WT, YAP−/−, and TAZ−/− hESCs. Scale bar, 75 μm. (F) G-banded karyotyping analysis of WT, YAP−/−, and TAZ−/− hESCs showing normal karyotypes. (G) Summary of the generated total and YAP/TAZ dKO clone numbers. The numerical data underlying this figure are included in S8 Data. Cas9, CRISPR associated protein 9 nuclease; CRISPR, Clustered Regularly Interspaced Short Palindromic Repeats; Ctrl, control; dKO, double knockout; ESC, embryonic stem cell; FOXA2, forkhead box A2; hESC, human embryonic stem cell; HR, homologous recombination; KO, knockout; RT-qPCR, reverse transcription quantitative polymerase chain reaction; SMA, smooth muscle actin; TAZ, transcriptional coactivator with PDZ-binding motif; TUJ1, beta-tubulin III; WT, wild type; YAP, Yes-associated protein. (TIF) [file pbio.3000201.s001.tif]

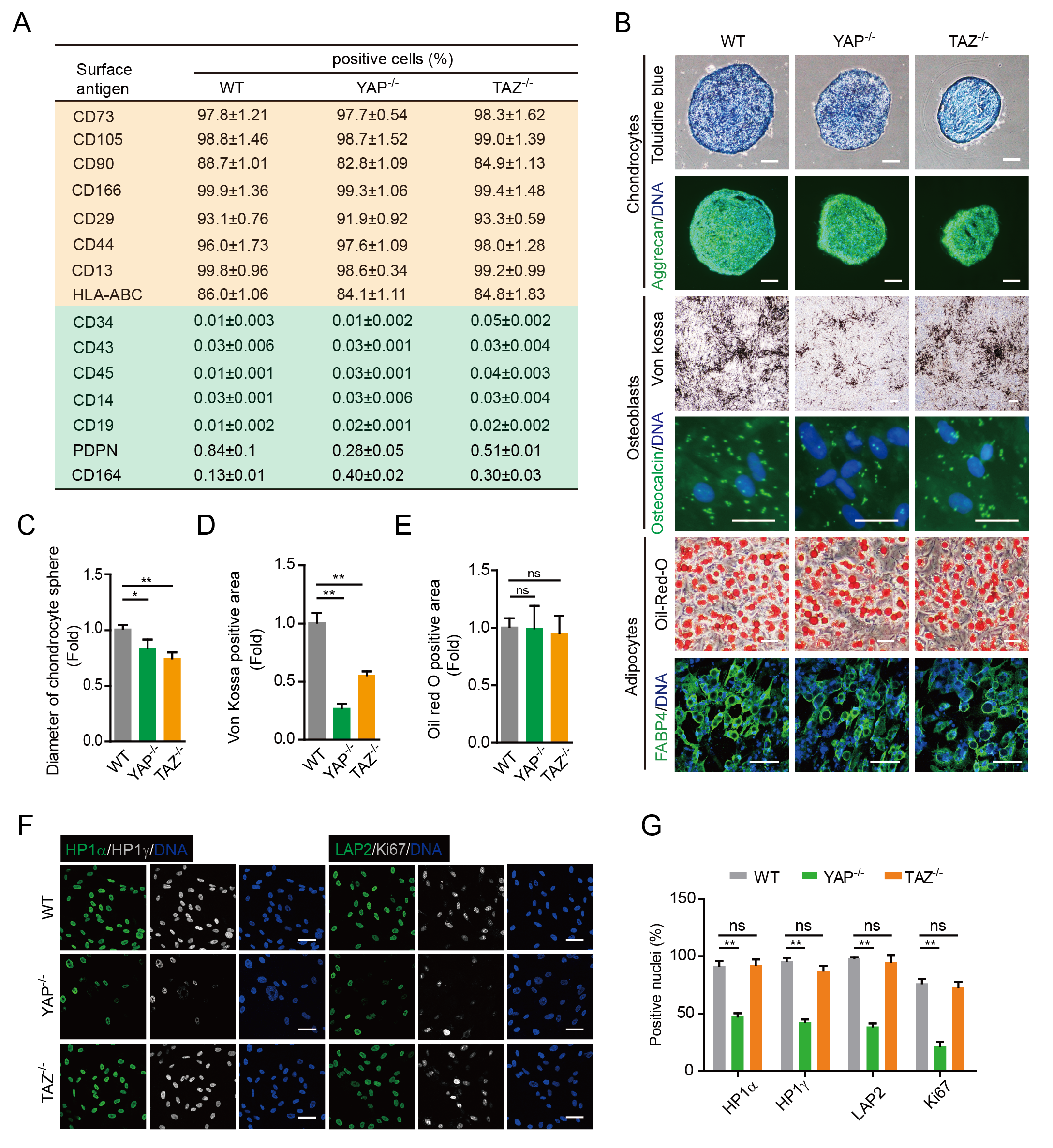

Supplement: S2 Fig — (A) Surface antigen expression levels of WT, YAP−/−, and TAZ−/− hMSCs. Data are presented as the mean ± SD, n = 3. (B) Characterization of the multilineage differentiation potential of WT, YAP−/−, and TAZ−/− hMSCs. Toluidine blue and Aggrecan staining were used to evaluate chondrogenesis. Von Kossa and Osteocalcin staining were used to evaluate osteogenesis. Oil Red O and FABP4 staining were used to evaluate adipogenesis. Scale bar, 25 μm. (C) The diameters of chondrocyte spheres were measured. Data are presented as the mean ± SD, n = 10, *P < 0.05, **P < 0.01. (D) Areas of Von Kossa–positive cells were calculated. Data are presented as the mean ± SD, n = 3, **P < 0.01. (E) Areas of Oil Red O-positive cells were calculated. Data are presented as the mean ± SD, n = 3. (F) Representative images of immunofluorescence staining for HP1α, HP1γ, LAP2, and Ki67 in hMSCs. Scale bar, 50 μm. (G) Quantification of HP1α−, HP1γ−, LAP2−, and Ki67-positive nuclei in WT, YAP−/−, and TAZ−/− hMSCs. More than 100 randomly selected nuclei were analyzed from each group. Data are presented as the mean ± SD, **P < 0.01. The numerical data underlying this figure are included in S8 Data. hMSC, human mesenchymal stem cell; HP1α, heterochromatin protein 1 alpha; HP1γ, heterochromatin protein 1 gamma; LAP2, lamina-associated protein 2; ns, not significant; PDPN, podoplanin; TAZ, transcriptional coactivator with PDZ-binding motif; WT, wild type; YAP, Yes-associated protein. (TIF) [file pbio.3000201.s002.tif]

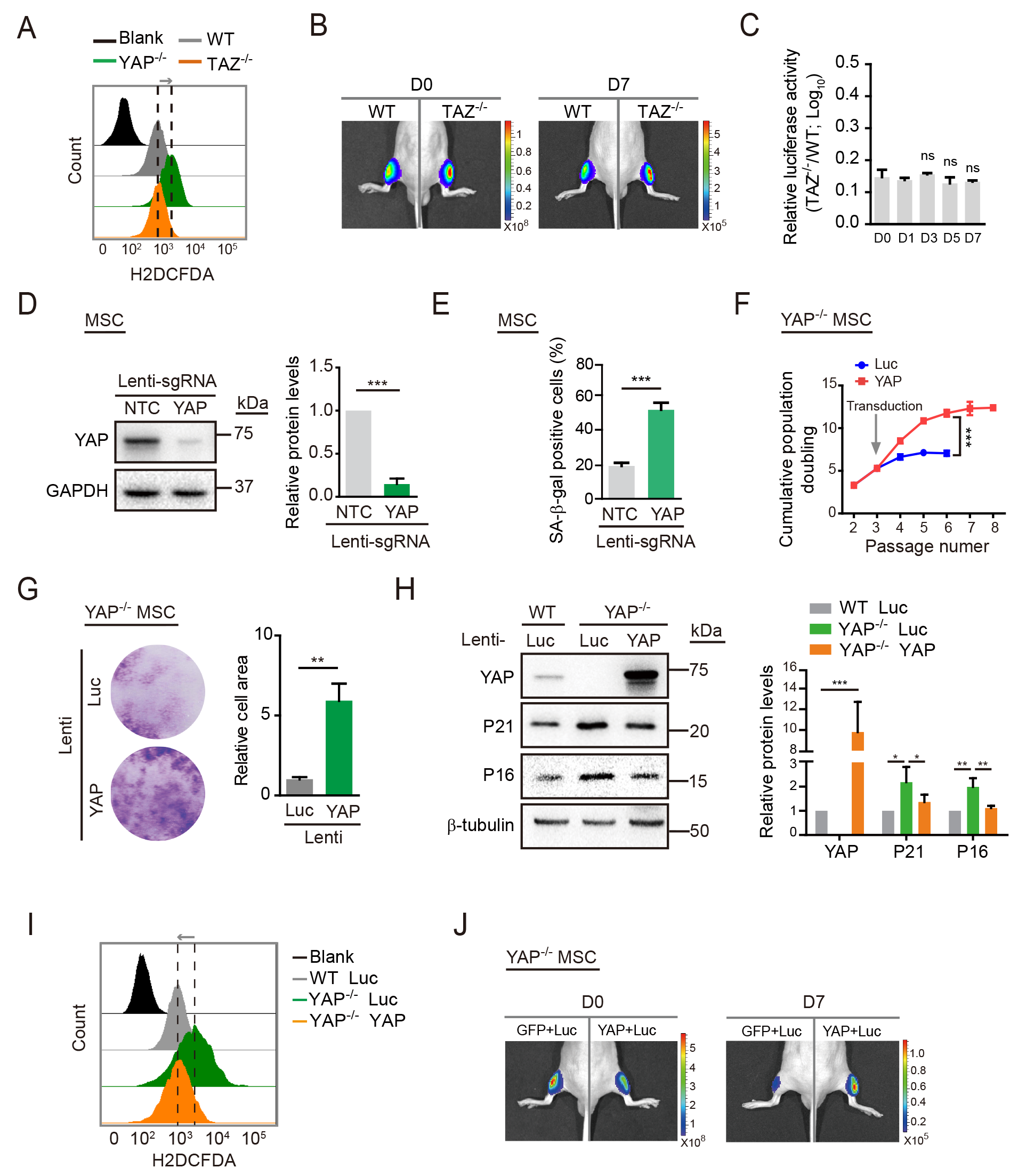

Supplement: S3 Fig — (A) Flow cytometry analysis of cellular ROS levels using H2DCFDA probes. (B) WT and TAZ−/− hMSCs were transduced with the lentivirus expressing Luc and injected into the TA muscle of immunodeficient mice. Luc activities were imaged at day 0, 1, 3, 5, and 7 after cell implantation. The representative images at day 0 and day 7 are shown. (C) The Luc activities were presented as the ratios of TAZ−/− to WT cells (log10 (fold)), mean ± SD, n = 5. (D) Western blot analysis of YAP in hMSCs transduced with lentiviruses expressing NTC or YAP sgRNA, as well as CRISPR/Cas9. GAPDH was used as a loading control (left). The protein levels normalized with GAPDH were shown as fold change relative to lenti-NTC–sgRNA–transduced hMSCs. Data are presented as the mean ± SD, n = 3, ***P < 0.001 (right). (E) SA-β-gal analysis of hMSCs transduced with lentiviruses expressing NTC or YAP sgRNA, as well as CRISPR/Cas9. Data are presented as the mean ± SD, n = 3, ***P < 0.001. (F) Cell growth curves of YAP−/− hMSCs transduced with a lentiviral vector encoding Luc or YAP. Data are presented as the mean ± SD, n = 3, **P < 0.01. (G) Analysis of the clonal expansion of YAP−/− hMSCs lentivirally expressing Luc or YAP. Areas of crystal violet–positive cells were calculated using ImageJ software. Data are presented as the mean ± SD, n = 3, ***P < 0.001. (H) Western blot analysis showing decreased expression of P16 and P21 upon the ectopic expression of YAP in YAP−/− hMSCs. β-tubulin was used as a loading control (left). The protein levels normalized with β-tubulin were shown as fold change relative to WT hMSCs. Data are presented as the mean ± SD, n = 3, *P < 0.05, **P < 0.01. (I) ROS detection in WT hMSCs transduced with the lentivirus expressing Luc and YAP−/− hMSCs transduced with lentiviruses expressing Luc or YAP. (J) YAP−/− hMSCs overexpressing GFP plus Luc and YAP−/− hMSCs overexpressing YAP plus Luc were implanted into the TA muscles of immunodeficient mice. Luc activities were imaged at day [file pbio.3000201.s003.tif]

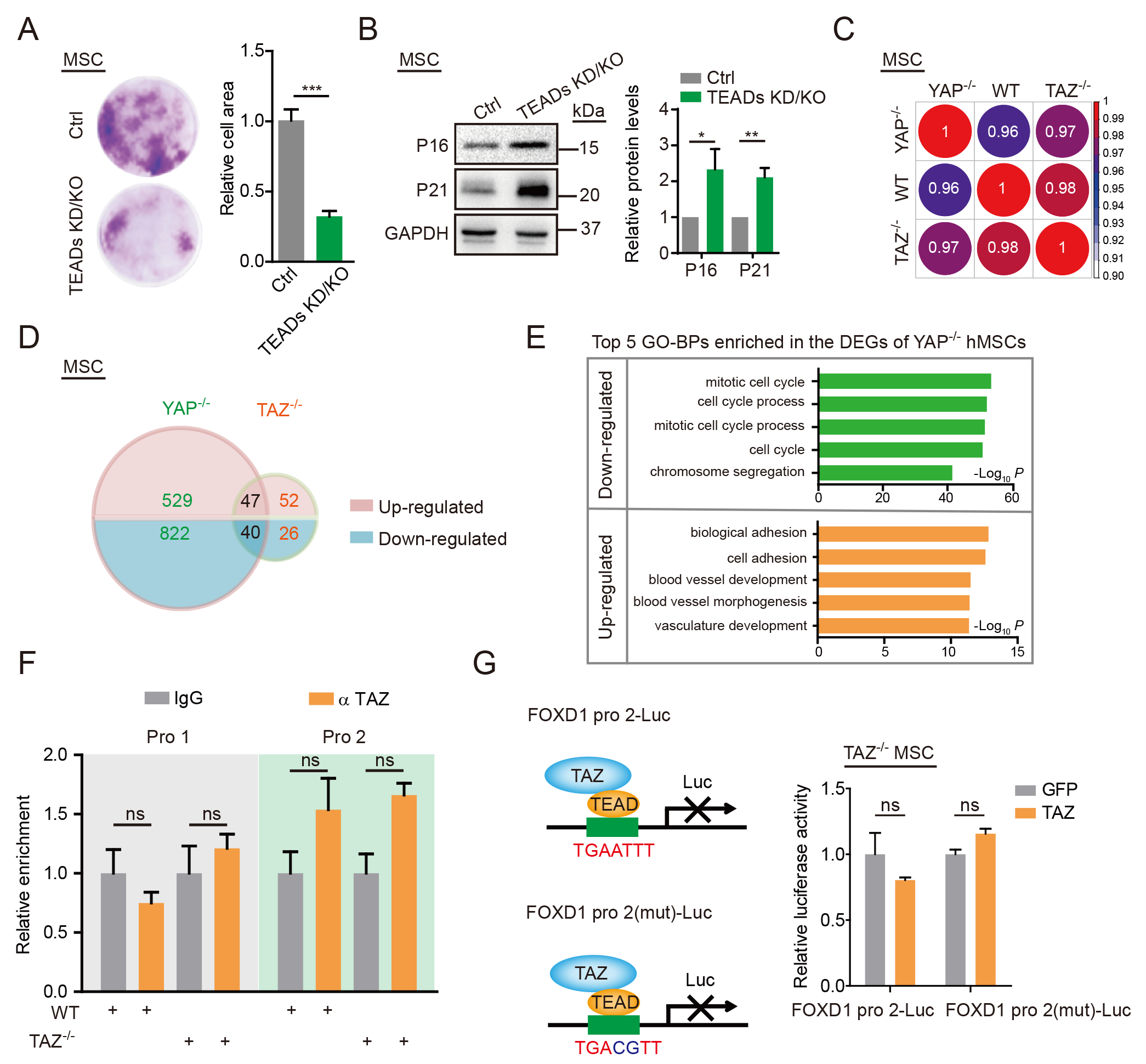

Supplement: S4 Fig — (A) Clonal expansion analysis of Ctrl and TEADs KD/KO hMSCs. Areas of crystal violet–positive cells were calculated using ImageJ software. Data are presented as the mean ± SD, n = 3, ***P < 0.001. (B) Western blots for P16 and P21 in Ctrl and TEADs KD/KO hMSCs. GAPDH was used as a loading control (left). The protein levels normalized with GAPDH were shown as fold change relative to Ctrl hMSCs. Data are presented as the mean ± SD, n = 3, *P < 0.05, **P < 0.01. (C) Pearson correlation coefficients for gene expression in WT, YAP−/−, and TAZ−/− hMSCs. (D) Venn diagrams showing DEGs in YAP−/− and TAZ−/− hMSCs relative to WT hMSCs. (E) The top 5 GO BPs enriched among the DEGs in YAP−/− hMSCs. (F) ChIP-qPCR for TAZ enrichment within different FOXD1 pro regions (Pro 1 and Pro 2) containing putative TEAD binding motifs. Data are presented as the mean ± SD, n = 3. (G) The FOXD1 pro containing the Pro 2 region and a mutation were cloned upstream of a Luc reporter, and the Luc activities were measured after transfection of GFP or TAZ. Data are presented as the mean ± SD, n = 3. The numerical data underlying this figure are included in S8 Data. BP, biological process; ChIP-qPCR, chromatin immunoprecipitation quantitative polymerase chain reaction; Ctrl, control; DEG, differentially expressed gene; FOXD1, forkhead box D1; GAPDH, glyceraldehyde-3-phosphate dehydrogenase; GFP, green fluorescent protein; GO, gene ontology; hMSC, human mesenchymal stem cell; KD, knockdown; KO, knockout; mut, mutant; ns, not significant; pro, promoter; TAZ, transcriptional coactivator with PDZ-binding motif; TEAD, TEA domain transcriptional factor; WT, wild type; YAP, Yes-associated protein. (TIF) [file pbio.3000201.s004.tif]

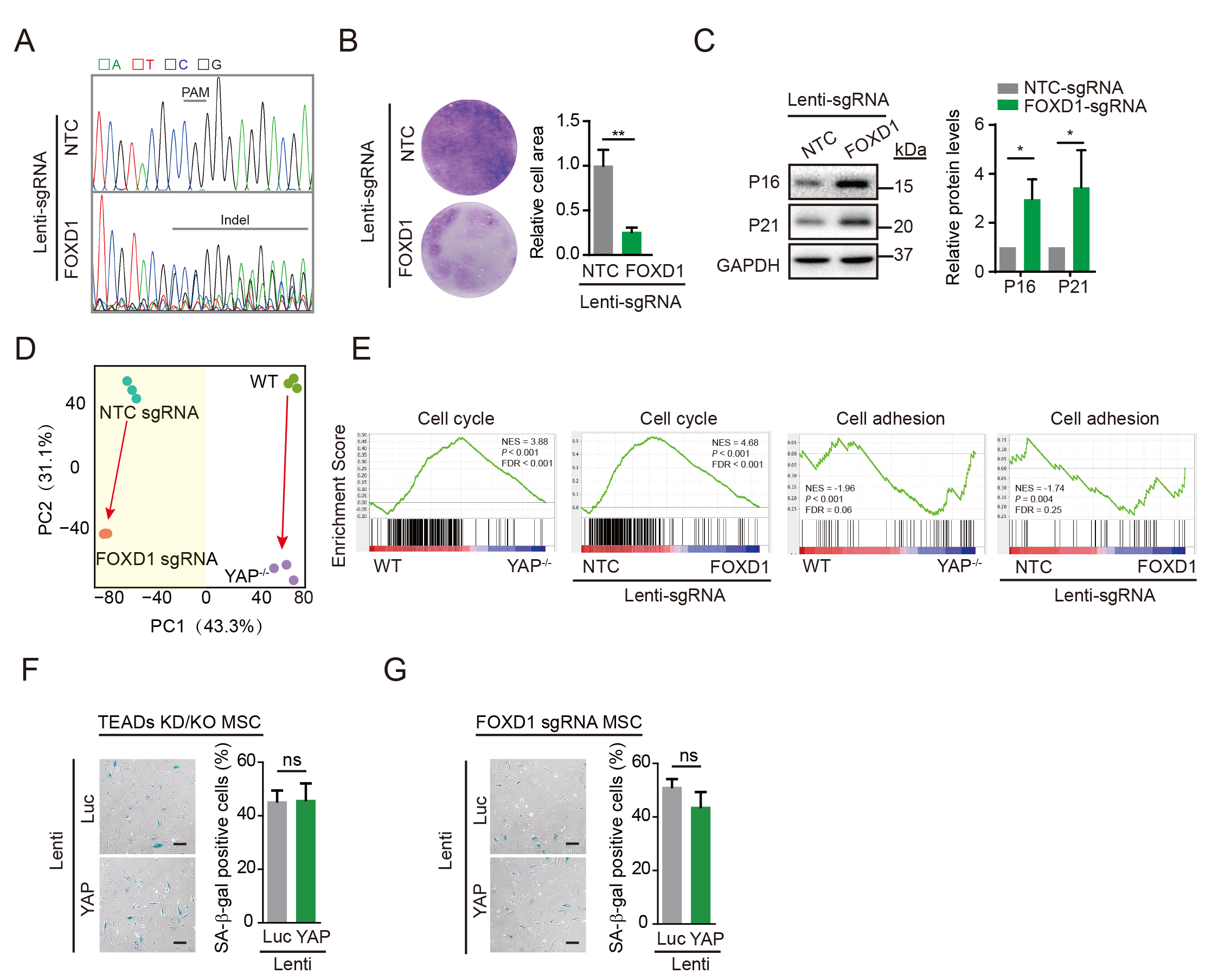

Supplement: S5 Fig — (A) Genomic sequencing of the FOXD1 locus in NTC and FOXD1 KO hMSCs. (B) Clonal expansion analysis of NTC and FOXD1 KO hMSCs. Areas of crystal violet–positive cells were calculated using ImageJ software. Data are presented as the mean ± SD, n = 3, **P < 0.01. (C) Western blot analysis for P16 and P21 in NTC and FOXD1 KO hMSCs. GAPDH was used as a loading control (left). The protein levels normalized with GAPDH were shown as fold change relative to NTC hMSCs. Data are presented as the mean ± SD, n = 3, *P < 0.05. (D) PC analysis of WT, YAP−/−, NTC, and FOXD1 KO hMSCs. (E) Comparison of the GSEA of cell cycle and cell adhesion genes between WT and YAP−/− hMSCs and between NTC and FOXD1 KO hMSCs. (F) SA-β-gal analysis of TEADs KD/KO hMSCs transduced with lentiviruses expressing Luc or YAP. Scale bar, 100 μm. Data are presented as the mean ± SD, n = 3. (G) SA-β-gal analysis of FOXD1 KO hMSCs transduced with lentiviruses expressing Luc or YAP. Scale bar, 100 μm. Data are presented as the mean ± SD, n = 3. The numerical data underlying this figure are included in S8 Data. FOXD1, forkhead box D1; GAPDH, glyceraldehyde-3-phosphate dehydrogenase; GSEA, Gene Set Enrichment Analysis; hMSC, human mesenchymal stem cell; Indel, insertion and deletion; KD, knockdown; KO, knockout; Luc, luciferase; ns, not significant; NTC, non-targeting control; PAM, protospacer-adjacent motif; PC, principal component; SA-β-gal, senescence-associated-β-galactosidase; TEAD, TEA domain transcriptional factor; WT, wild type; YAP, Yes-associated protein. (TIF) [file pbio.3000201.s005.tif]

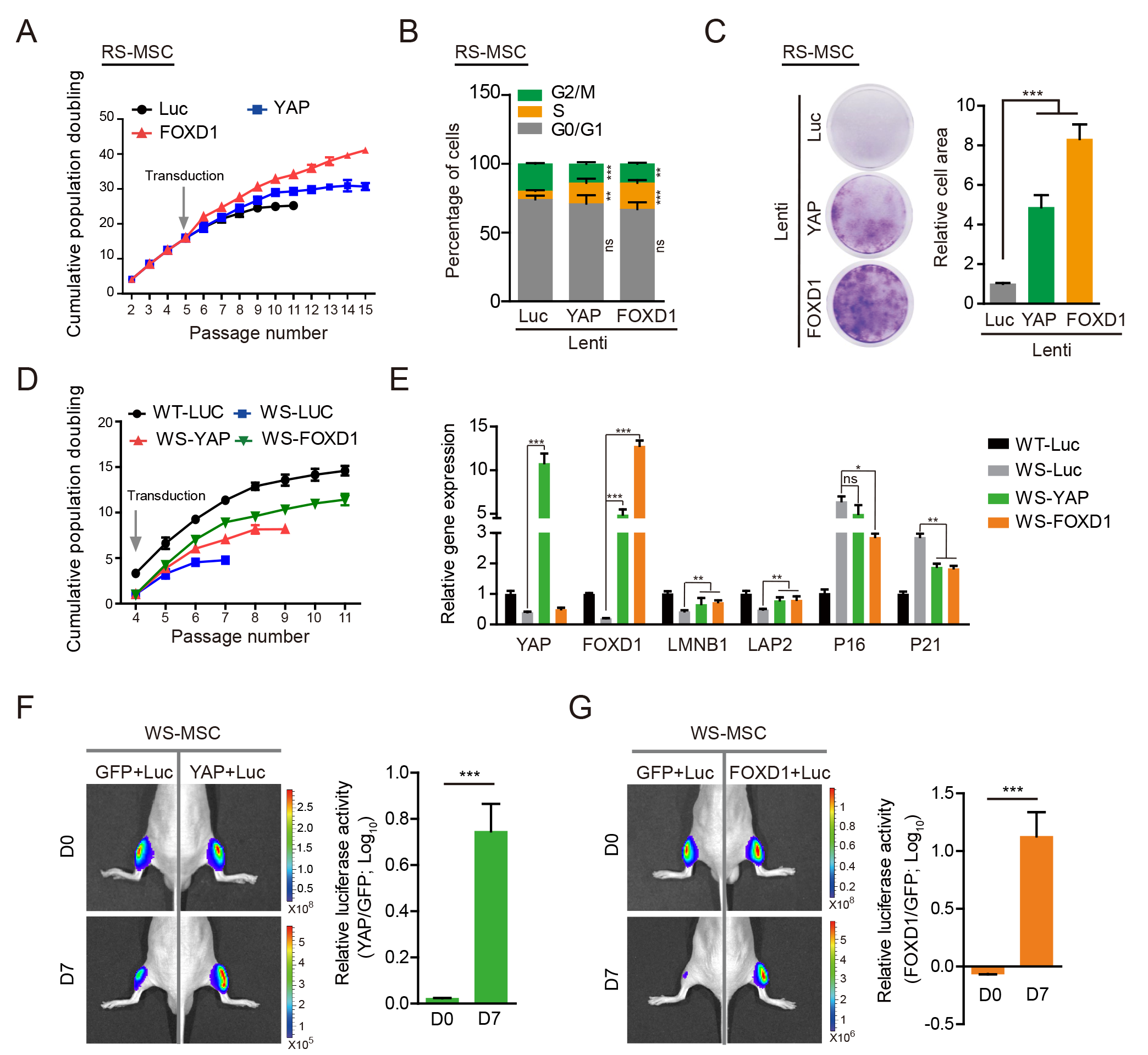

Supplement: S6 Fig — (A) Growth curve of RS hMSCs transduced with lentiviruses expressing Luc, YAP, or FOXD1. (B) Cell cycle analysis of RS hMSCs transduced with lentiviruses expressing Luc, YAP, or FOXD1. Data are presented as the mean ± SD, n = 3, **P < 0.01, ***P < 0.001. (C) Clonal expansion abilities of RS hMSCs transduced with lentiviruses expressing Luc, YAP, or FOXD1. Data are presented as the mean ± SD, n = 3, ***P < 0.001. (D) Growth curves of WS hMSCs transduced with lentiviruses expressing Luc, YAP, or FOXD1. (E) RT-qPCR of aging-associated markers in WS hMSCs transduced with lentiviruses expressing Luc, YAP, or FOXD1. Data are presented as the mean ± SD, n = 3, *P < 0.05, **P < 0.01, ***P < 0.001. (F) Measurement of Luc activity using IVIS at day 0 and day 7 after in vivo implantation of WS hMSCs transduced with lentiviruses expressing YAP or GFP. Data are presented as the ratios of YAP to GFP (log10 (fold)), mean ± SD, n = 3, ***P < 0.001. (G) Measurement of Luc activity using IVIS at day 0 and day 7 after in vivo implantation of WS hMSCs transduced with lentiviruses expressing FOXD1 or GFP. Data are presented as the ratios of FOXD1 to GFP (log10 (fold)), mean ± SD, n = 3, ***P < 0.001. The numerical data underlying this figure are included in S8 Data. FOXD1, forkhead box D1; GFP, green fluorescent protein; hMSC, human mesenchymal stem cell; IVIS, in vivo imaging system; LAP2, lamina-associated protein 2; LMNB1, Lamin B1; Luc, luciferase; ns, not significant; RS, replicative-senescent; RT-qPCR, reverse transcription quantitative polymerase chain reaction; WS, Werner syndrome; YAP, Yes-associated protein. (TIF) [file pbio.3000201.s006.tif]

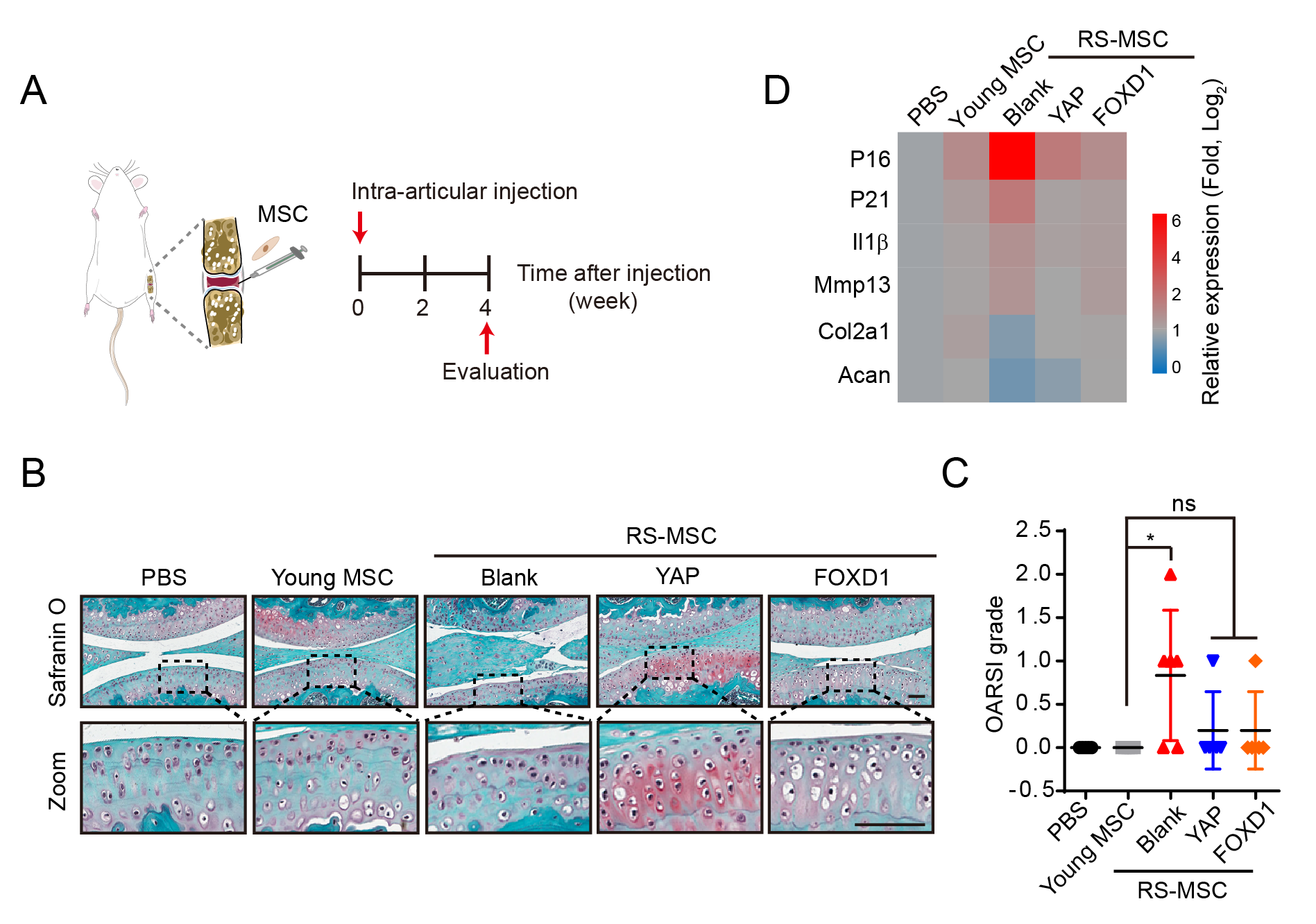

Supplement: S7 Fig — (A) Schematic of the time course for experiments in B–D. (B) Safranin O and Fast Green staining of articular cartilage from mice that transplanted with indicated hMSCs. Representative images are shown. Scale bar, 100 μm. (C) OARSI scores of articular joints (PBS, n = 5; young hMSCs, n = 5; RS hMSCs, n = 6; RS hMSCs overexpressing YAP, n = 5; RS hMSCs overexpressing FOXD1, n = 5). Data are presented as the mean ± SD, *P < 0.05. (D) Heat map showing RT-qPCR analysis of the indicated genes associated with senescence, inflammation and chondrogenesis in joints transplanted with indicated hMSCs. Expression levels of the indicated genes were normalized to PBS group. The numerical data underlying this figure are included in S8 Data. Acan, aggrecan; Col2a1, collagen, type II, alpha 1; FOXD1, forkhead box D1; hMSC, human mesenchymal stem cell; Il1β, interleukin 1 beta; Mmp13, matrix metallopeptidase 13; ns, not significant; OARSI, Osteoarthritis Research Society International; RS, replicative-senescent; RT-qPCR, reverse transcription quantitative polymerase chain reaction; YAP, Yes-associated protein. (TIF) [file pbio.3000201.s007.tif]

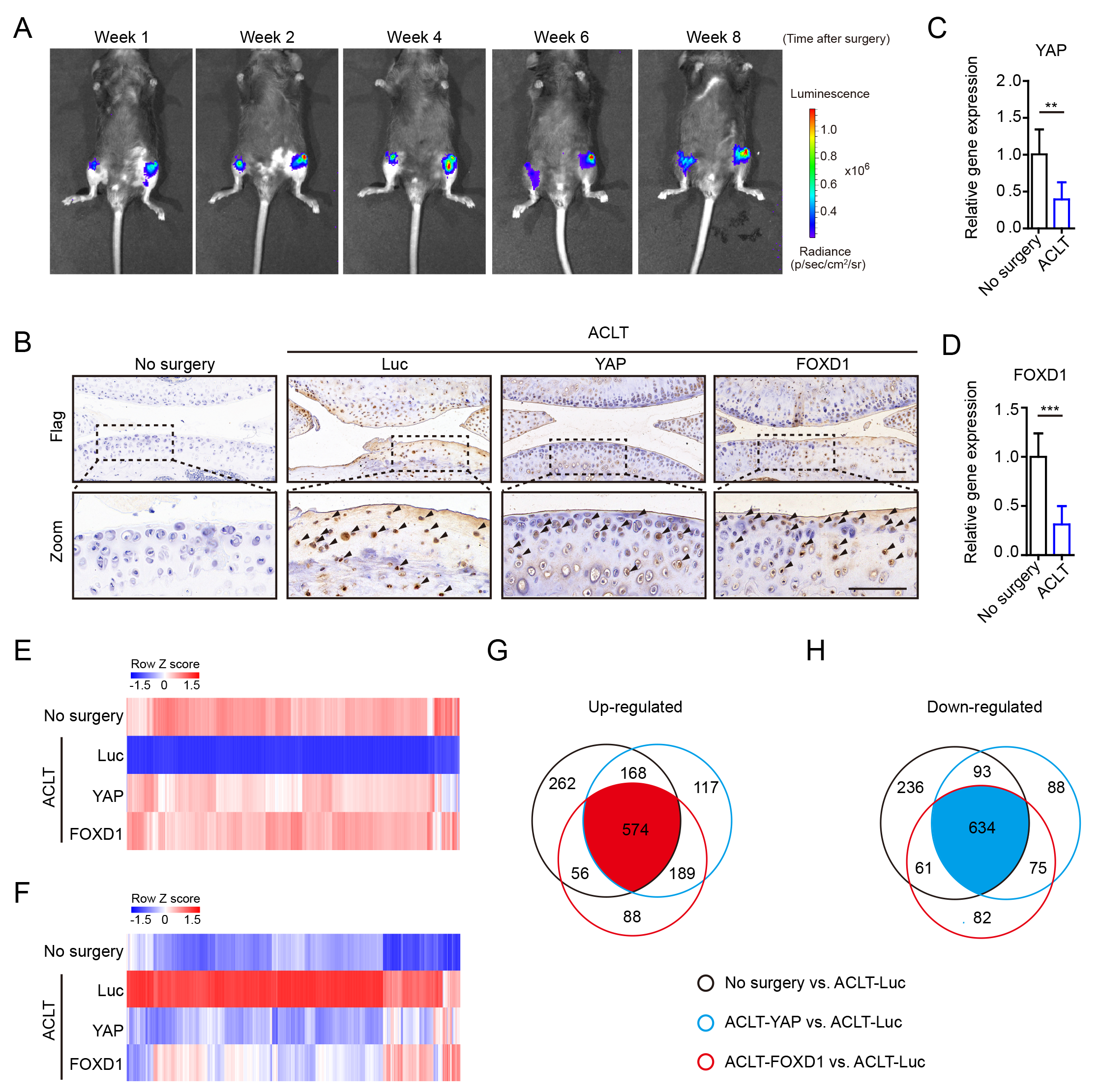

Supplement: S8 Fig — (A) Detection of the luminescence in the joints at different time points after injection of the lentivirus expressing Luc intra-articularly. The representative images are shown. (B) Immunohistochemical staining of indicated flag-tagged proteins in the joint cartilage of mice that did not receive surgery and of ACLT mice treated with lentiviruses expressing flag-tagged Luc, YAP, or FOXD1. Scale bar, 50 μm. (C and D) RT-qPCR analysis of YAP and FOXD1 expression in the joints of mice that did not undergo surgery (n = 6) and ACLT (n = 8). Data are presented as the mean ± SD, **P < 0.01, ***P < 0.001. (E and F) Heat maps showing relative mRNA expression levels of the differentially expressed genes in the ACLT-Luc group compared to the joints that did not undergo surgery. Genes were sorted by the fold change and P value (fold change > 2 or < 0.5, P < 0.01). Corresponding gene expression profiles obtained from ACLT-YAP and ACLT-FOXD1 groups are also shown. (G and H) Venn diagrams showing differentially expressed genes (no surgery versus ACLT-Luc; ACLT-YAP versus ACLT-Luc; ACLT-FOXD1 versus ACLT-Luc). The numerical data underlying this figure are included in S8 Data. ACLT, anterior cruciate ligament transection; FOXD1, forkhead box D1; Luc, luciferase; RT-qPCR, reverse transcription quantitative polymerase chain reaction; YAP, Yes-associated protein. (TIF) [file pbio.3000201.s008.tif]
